# Has the manufacturing policy helped to promote the logistics industry?

**DOI:** 10.1371/journal.pone.0235292

**Published:** 2020-07-02

**Authors:** Dan He, Jialiang Yang, Zhengming Wang, Wenchao Li

**Affiliations:** School of Finance and Economics, Jiangsu University, Zhenjiang City, Jiangsu Province, China; Institute for Advanced Sustainability Studies, GERMANY

## Abstract

The logistics industry is a derivative industry of manufacturing services extraposition. A variety of strategies to develop the manufacturing industry are important programs of action for China's manufacturing strategic power, and it is of great significance to promote the high-quality development of the logistics industry. This paper takes strong manufacturing provinces with the development of the logistics industry as the research object and applies network DEA measuring the production efficiency and service efficiency of the logistics industry from 2004 to 2017. This paper adopts the “Made in China 2025” strategy as a natural experiment and uses double difference to study the impact of manufacturing policies on the high-quality development of the logistics industry. The empirical results show that compared with the Reference group, the impact of the “Made in China 2025” strategy led to a significant increase in the production efficiency and service efficiency of the experimental group. The group-based test based on innovation type shows that independent innovation has a significant positive effect on the high-quality development of the logistics industry, which shows that from the perspective of technological innovation, independent innovation is the main path of the “Made in China 2025” strategy to promote the high-quality development of the logistics industry. This paper not only identifies the causal relationship between the “Made in China 2025” strategy and the high-quality development of the logistics industry but also helps clarify the mechanism of how manufacturing policies improve the high-quality development of the logistics industry, which has important implications for further promoting the combined development between manufacturing and logistics.

## Introduction

A variety of strategies for developing the manufacturing industry are action programs for China to break through the dual squeeze of advanced technologies in developed countries and low-cost competition in developing countries, to actively adapt its global industrial reform and development trend and reshape its competitive advantage in the manufacturing industry [1]. Logistics industry reforms benefited both Foreign Direct Investment (FDI) and locally owned manufacturing firms [2, 3]. The logistics industry is a derivative industry generated by the outsourcing of services in the process of the breakdown and specialization of the manufacturing industry. This industry has a natural co-evolution relationship with the manufacturing industry.

In recent years, driven by the demand of the manufacturing industry, China's logistics industry has achieved significant improvements in infrastructure conditions, policy environment, and service functions. The total amount of social logistics has continued to increase, reaching 252.8 trillion yuan in 2017, which was four times higher in 2007 than in 2005. Calculated at comparable prices, the average annual growth rate is approximately 12%, and the proportion of the added value of the logistics industry to gross domestic product (GDP) increased from 6.7% in 2005 to approximately 7.4% in 2017. We observe that at the stage of the comprehensive promotion of China's manufacturing industry from big to strong, the logistics industry is undoubtedly a fundamental, strategically important industry to ensure sustainable economic development. However, the inherent characteristics of the logistics industry, with transportation as its main body, determine its basic characteristics of high energy dependence.

China's logistics industry is in the initial stage of transformation in scale expansion and has not eliminated the development mode of high energy consumption and high pollution. In terms of total volume, the logistics industry is one of China’s major energy-consuming industries. *The China Statistical Yearbook (2018)* shows that in 2016, the energy consumption of the logistics industry accounted for 9.10% of the total energy consumption of various industries in the country, and its consumption of refined oil ranks first among all industries in the country. Nearly 46.44% of gasoline and 65.73% of diesel are consumed by the logistics industry. In terms of growth rate, the logistics industry is one of China's fastest-growing energy consumption industries [4, 5]: Its average annual growth rate is over 8%, and this figure is 2 percentage points higher than the average annual growth rate of energy consumption in the whole society. Notably, the carbon emissions of agriculture, industry, construction, commerce, and other industries have declined to varying degrees, but the carbon emission intensity of the logistics industry has continued to increase [6, 7], which is higher than the average annual growth rate of various industries in the country [8]. Therefore, the high-quality development of the logistics industry is of great significance to the transformation and upgrading of China's manufacturing industry.

Based on our review of the literature, the interactive relationship between logistics and manufacturing has been affirmed. The development of the manufacturing industry still requires the support of the logistics industry, and some studies have shown that regional legal system can promote the positive impact of technological innovation on brand equity [9]. But will the "Made in China 2025" strategy promote industrial high-quality development of China's logistics industry while promoting industrial innovation and green manufacturing? Under the requirements of the current economic development and the coordinated development of the two industries, the development of the logistics industry cannot continue its previous extensive development path, and new requirements must be implemented for the development of the logistics industry.

Studies have started a rich discussion on the coordinated development relationship between manufacturing and logistics, clarified the inevitable trend of the combined development of manufacturing and logistics, and fully affirmed the important role of manufacturing transformation and upgrading to the transformation and upgrading of the logistics industry; however, research on the development of the logistics industry from the perspective of manufacturing policy has been rare. The transformation and upgrading of logistics enterprises is an inevitable product of manufacturing servitization: It not only depends on the development of manufacturing industries but also promotes the transformation and upgrading of manufacturing enterprises; additionally, it has the significant characteristics of industrial linkage and coordinated development and demonstrates the general trend of integration and development in the development of higher-level industries [10]. Generally, the combined development of the manufacturing industry and the logistics industry refers to the integration of manufacturing enterprises’ logistics business and logistics enterprises’ logistics operations, based on the industrial relationship between the manufacturing industry and the logistics industry [11].

Wu Xiaoyan and Lu Shichang (2018) also conduct an empirical analysis of the coordinated development of the logistics industry and the three industries. The results show that the degree of industrial synergy between the secondary industry and the logistics industry has a greater impact on the orderliness of the development of the logistics industry. In particular, the transformation and upgrading of the manufacturing industry require a higher level of logistics supply. The deep integration of the two industries can effectively promote the flow and diffusion of factors between industries [12]. However, the integrated development model of manufacturing and the logistics industry is not simply adding services on the basis of manufacturing products; the basic activities of logistics include warehousing, distribution processing, and transportation, which are integrated into the manufacturing industry in an embedded manner. The coordinated development of the manufacturing industry and logistics industry can realize the back-feeding function of the logistics industry, clarify the difference in R&D efficiency between the two industries. So that the service industry continues to intervene in manufacturing through research and development, and promoting the integration of the two industries [13, 14].

Wang Jun and Cao Lixin (2012) proposed that the combined development of manufacturing and logistics is necessary for manufacturing companies to reduce costs, enhance competitiveness, and transfer risks. However, the policy environment for combined development of the two industries in China is not perfect, and the quality of logistics services of most logistics companies cannot fulfill the needs of manufacturing and is developing slowly [15]. We observe that the high-quality development of the logistics industry requires not only the synergistic effect of the manufacturing industry but also a sound, conducive policy environment. The "Made in China 2025" strategy, proposed by China in 2015, has undoubtedly provided unprecedented opportunities. Thus, this paper uses the strong manufacturing province as a carrier to study the impact of the "Made in China 2025" strategy on the high-quality development of the logistics industry. This paper also considers that the technological innovation element is the main boosting path of this impact [16]. Relevant research has found that the introduction and leveraging the best technologies may yield competitive advantages and higher financial rewards for Logistics Service Providers [17]. Both political and business guanxi have a positive effect on logistics service innovation [18]. and from the perspective of technological innovation, explore the effect of the "Made in China 2025" strategy on the high-quality development of the logistics industry.

This paper makes the following contributions to the literature: (1) Through the network DEA, the operation process of the logistics industry is divided into production processes and service processes [19, 20]. It goes beyond the "black box" handling disadvantages of efficiency measurement we observe in most of the literature and is closer to the network-based operation form of the logistics industry. We use two paragraphs, for efficiency, to describe the high-quality development of the logistics industry and enrich the related theoretical research on the high-quality development of the logistics industry. (2) In the theory of structural changes and high-quality development in China, industrialization is not the same as the industrialization of manufacturing [21]. Industrialization is not simply understood as the industrialization of the manufacturing industry. The manufacturing industry and other major related industries should be considered together to achieve comprehensive structural upgrades and high-quality development. Therefore, this paper uses a strong manufacturing province as a carrier to verify the promotion effect of the "Made in China 2025" strategy on the high-quality development of the logistics industry and—to a certain extent—enrich the relevant research on high-quality development.

Technical innovation factors are the main factors that drive the transformation and upgrading of the logistics industry [22]. In addition to verifying that R&D and innovation are important paths for industrial upgrading, this paper also makes a reasonable distinction between this path (independent innovation, imitation innovation, direct introduction innovation, and indirect introduction innovation) to verify which analyzes the path of more specific technological innovation to promote the high-quality development of the logistics industry.

Therefore, previous studies have analyzed almost every perspective of the collaborative development between manufacturing industry and logistics industry. But, rarely about impact of manufacturing policies on the logistics industry. This paper chooses "Made in China 2025" strategy to present manufacturing policy, and tests its impact on the high-quality development of logistics industry. And then, this paper discusses the effective paths to promote the impact from the perspective of technological innovation.

## Model building

The logistics industry is a collection of activities that organically integrate basic functions, such as transportation, storage, and distribution processing, according to actual needs. Therefore, the logistics industry operation process can be divided into production processes and service processes; thus, the logistics industry can be regarded as a typical multiactivity and multistage industry. Its decision-making unit has a network production structure, and the efficiency of different stages differs. Based on the aforementioned considerations, this paper chooses the network DEA model to evaluate the high-quality development of the logistics industry in each region.

### Network DEA

**Fig 1:**
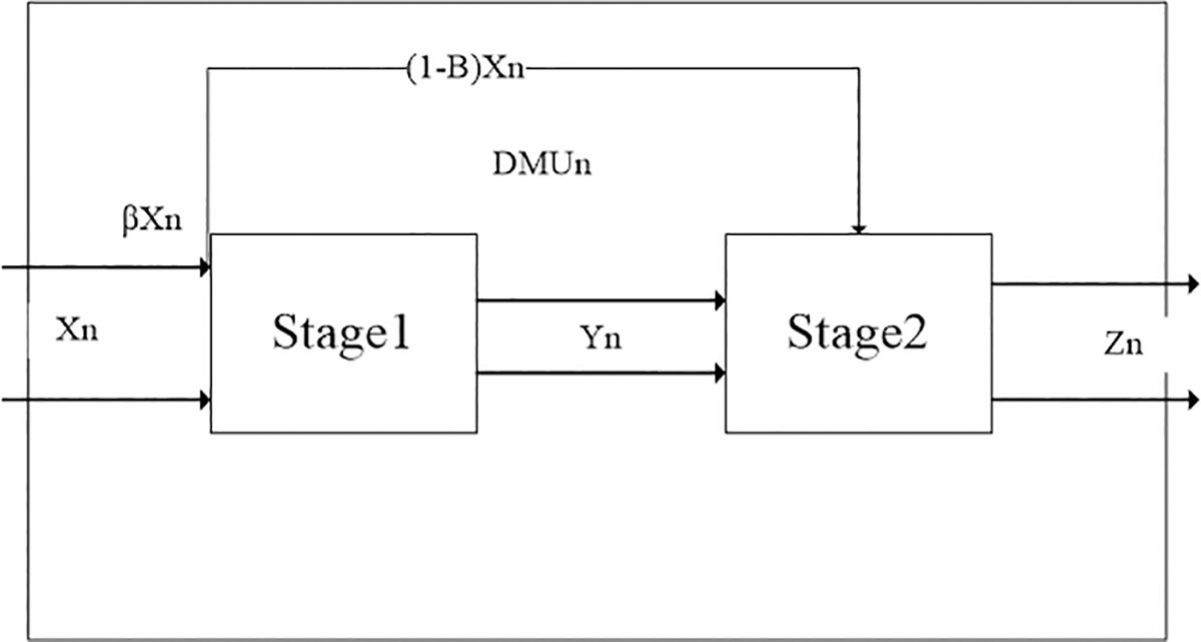
Modeling process of network DEA model.

In this paper, two-stage network DEA model is used to measure the efficiency of high-quality development of logistics industry. The first and second stage network DEA model can open the "black box" of production system and evaluate the logistics industry high-quality development. According to the characteristics of input and output in each stage, this paper defines the efficiency of the first stage as the production efficiency of logistics industry high-quality development, and the efficiency of the second stage as the R&D efficiency of high-quality development of logistics industry. The idea of two-stage network DEA model is shown in the figure below [23]:

According to Fig 1, there are *n* decision-making units in the production system, Namely *DMU*, In stage 1, the quantity of input indicators is *L*, and the quantity of intermediate output indicators is *M*. The input indicators in the first stage will participate in the production in the second stage in a certain proportion. Therefore, input indicators of stage 2 include *L*+*M* items and output indicators are *O* items. *X*_ln_ is the l^th^ quantity of input indicators of the n^th^ decision-making unit in the first stage (*X*_ln_>0,*n* = 1,2,…,*N*,*l* = 1,2,…,*L*), *a*_*l*_ is the weight corresponding to *X*_ln_(*l* = 1,2,…,*L*), *y*_*mn*_ is the intermediate output, that is, the m^th^ output quantity of the nth *DMU* in stage 1 (*y*_*mn*_>0,*m* = 1,2,…,*M*,*n* = 1,2,…,*N*). *b*_*m*_ is the weight corresponding to *y*_*mn*_(*m* = 1,2,…,*M*). *Z*_on_ is the o^th^ quantity of output indicators of the nth decision-making unit in the second stage (*Z*_*on*_>0,*o* = 1,2,…,*O*,*n* = 1,2,…,*N*), *c*_*o*_ is the weight corresponding to *Z*_*on*_(*o* = 1,2,…,*O*). *β*_*l*_ is the weight of input quantity in stage 1, 1-*β*_*l*_ is the weight of input quantity in stage 2(*β*_*l*_>0,*l* = 1,2,…,*L*). Record as formula (1):

$$\{\begin{array}{l} X_{n}={\left(x_{1n}\ldots x_{ln}\ldots x_{Ln}\right)}^{T},a={\left(a_{1}\ldots a_{2}\ldots a_{L}\right)}^{T}\\ Y_{n}={\left(y_{1n}\ldots y_{mn}\ldots x_{Mn}\right)}^{T},b={\left(b_{1}\ldots b_{2}\ldots b_{L}\right)}^{T}\\ Z_{n}={\left(z_{1n}\ldots z_{ln}\ldots z_{On}\right)}^{T},a={\left(c_{1}\ldots c_{2}\ldots c_{O}\right)}^{T}\\ \beta ={\left(\beta_{1}\ldots \beta_{l}\ldots \beta_{L}\right)}^{T},e={\left(1\ldots 1\ldots 1\right)}^{T} \end{array}$$ (1)

*X*_*n*_, *Y*_*n*_ and *Z*_*n*_ are input vector, intermediate output vector and final output vector of the n^th^ *DMU* respectively, *a*, *b* and *c* are the corresponding weight vectors. *β* represents the proportion weight vector of input quantity in stage 2 of input indicators in stage 1. The two-stage network DEA model divides the input process of *DMU*_*n*_ into two stages: *β*•*X*_*n*_ represents the input quantity of input indicators in stage 1. (*e*−*β*)•*X*_*n*_ indicates that partial output of the input indicators in stage 1 is added to the productive process in stage 2. The weight vector of input-output indicators in stage 1 is *a*∈*E*^*L*^,*b*∈*E*^*M*^, The efficiency evaluation expression and constraint conditions of *DMU*_*n*_ in stage 1 are as follows (2):

$$\left\{ \begin{array}{l} \max\frac{b^{T}Y_{0}}{a^{T}(\beta •X_{0})}\\ s.t.\left\{ \begin{array}{l} \frac{b^{T}Y_{n}}{a_{T}(\beta •X_{n})}\leq 1,n\in N\\ 0\leq \beta \leq e,a\geq 0,b\geq 0,\beta •a\neq 0 \end{array}\right. \end{array}\right.$$ (2)

The weight vector of input-output indicators in stage 2 is *a*∈*E*^*L*^,*b*∈*E*^*M*^,*c*∈*E*^*O*^, the efficiency evaluation expression and constraint conditions of *DMU*_*n*_ in stage 2 are as follows (3):

$$\left\{ \begin{array}{l} \max\frac{c^{T}Z_{0}}{a^{T}((e-\beta )•X_{0})+b^{T}Y_{0}}\\ s.t.\left\{ \begin{array}{l} \frac{c^{T}Z_{n}}{a_{T}((e-\beta )•X_{n})+b^{T}Y_{0}}\leq 1,n\in N\\ 0\leq \beta \leq e,a\geq 0,b\geq 0,c\geq 0,\beta •(e-a)\neq 0 \end{array}\right. \end{array}\right.$$ (3)

The linear programming equations of formulas (2) and (3), are (4) and (5) respectively.

$$\left\{ \begin{array}{l} \max\varepsilon_{1}^{T}Y_{0}\\ s.t.\left\{ \begin{array}{l} \varphi_{1}^{T}(\beta •X_{n})-\varepsilon_{1}^{T}Y_{n}\geq 0,n\in N\\ \varphi_{1}^{T}(\beta •X_{0})=1\\ 0\leq \beta \leq e,\varepsilon_{1}\geq 0,\varphi_{1}\geq 0 \end{array}\right. \end{array}\right.$$ (4)

$$\left\{ \begin{array}{l} \max\sigma_{2}^{T}Z_{0}\\ s.t.\left\{ \begin{array}{l} \varphi_{2}^{T}((e-\beta )•X_{n})+\varepsilon_{2}^{T}Y_{n}-\sigma_{2}^{T}Z_{n}\geq 0,n\in N\\ \varphi_{2}^{T}((e-\beta )•X_{n})+\varepsilon_{2}^{T}Y_{n}=1\\ 0\leq \beta \leq e,\varepsilon_{2}\geq 0,\varphi_{2}\geq 0,\sigma_{2}\geq 0 \end{array}\right. \end{array}\right.$$ (5)

By integrating formula (4) and (5), the total efficiency under comprehensive consideration can be obtained, such as formula (6):

$$\left\{ \begin{array}{l} \max\left\{\beta •\frac{b^{T}Y_{0}}{a^{T}(\beta •X_{0})}+(1-\beta )•\frac{c^{T}Z_{0}}{a^{T}((e-\beta )•X_{0})+b^{T}Y_{0}}\right\}\\ s.t.\left\{ \begin{array}{l} \frac{b^{T}Y_{n}}{a^{T}(\beta •X_{0})}\leq 1,n\in N\\ \frac{c^{T}Z_{n}}{a^{T}((e-\beta )•X_{n})+b^{T}Y_{n}}\leq 1,n\in N\\ 0\leq \beta \leq e,a\geq 0,b\geq 0,c\geq 0\\ \beta •a\neq 0,\beta •(e-a)\neq 0 \end{array}\right. \end{array}\right.$$ (6)

### Difference-in-difference model (DID)

The DID model is a popular policy evaluation model based on quasi-natural experiments in the academic community.

The model can mitigate the impact of other factors on the assessment results through two better differential policies. In the DID model, the most critical factors are the time of policy shock and the selection of the Study group and Reference group.

The policy time of this paper is 2015 because in December 2014, the concept of "Made in China 2025" was first proposed. On May 19, 2015, the State Council formally issued "Made in China 2025." The strategy has officially entered the stage of practical progress. The "Made in China 2025" strategy can be regarded as a quasi-natural experiment. In the long run, the strategy will promote the overall logistics industry in China. However, in the short term, the strategy will have a greater impact on the development of the logistics industry as a demonstration region in the country because in these places, it is more conducive to mobilizing local initiative and creativity, exploring new models and paths of manufacturing transformation and upgrading under the new normal. This provides the basis for the processing group and Reference group in this paper. In this paper, the provinces containing the exemplary cities are treated as the processing group and the remainder are set as the Reference group because the demonstration areas can be promoted through demonstration; then, we prioritize the realization of the province's logistics industry to improve the quality and efficiency, from big to strong.

Based on the design of the benchmark model, this paper designs the following formula (7) model to identify the impact of the "Made in China 2025" strategy on the high-quality development of the logistics industry:

$$HQD_{it}=\theta (id_{it}\times time_{it})+\alpha x_{it}+\lambda_{t}+\mu_{i}+yeartrend_{i}+\varepsilon_{it}$$ (7)

Among them, i and t represent regions and years, respectively; HQD represents the high-quality development level of the logistics industry, specifically expressed by the two-stage efficiency (*tfp1*, *tfp2*) measured by the network DEA, and replaced by the total efficiency (*tfp*) in the robustness test; a regional grouping variable with an id equal to 1 is the processing group, and equal to 0 is the Reference group; time is the time grouping variable, the value is 2004–2014 is 0, and the value is 2015–2017. On behalf of the control variable group, according to related literature [24, 25], this paper uses the scale of economic growth, retail sales of social consumer goods, fixed assets, industrial structure, government influence, and the average wage of logistics industry employees as the control variables. And represent fixed time effects and individual fixed effects, respectively, and double fixed effects of individuals and time. In addition, this paper also must identify the role and changing trend of the "Made in China 2025" strategy for the high-quality development of the logistics industry each year; thus, the following model is established (8):

$$HQD_{it}==\sum_{t=2015}^{t=2017}\theta (id_{it}\times {\mathrm{year}}_{t})+\alpha x_{it}+\lambda_{t}+\mu_{i}+yeartrend_{i}+\varepsilon_{it}$$ (8)

*year*_*t*_ in Eq (8) represents the dummy variable of the year, and the values are 2015, 2016, and 2017. The coefficients *θ* are indicators that reflect the effects and trends of the “Made in China 2025” strategy on the high-quality development of the Chinese logistics industry.

## Variable selection and data description

The concept of a "manufacturing strong province" in this paper is based on Professor Li Lianshui's follow-up research on China's manufacturing industry. According to China's economic development trends and requirements, by constructing an index system that includes economic creativity, scientific and technological innovation, energy-intensive capabilities, environmental protection capabilities, and social contribution capabilities, we select Jiangsu, Guangdong, Shandong, Zhejiang, Shanghai, Tianjin, Anhui, Beijing, Hunan, and Chongqing as the research target [26]. As an emerging modern service industry, the industry definition of "logistics industry" is gradually becoming clear, but statistics on the "logistics industry" have rarely been published. However, some yearbooks, such as "China Tertiary Industry Statistical Yearbook" and "China Logistics Yearbook," have considered the logistics industry as an independent category, but the relevant data remains insufficient. From the statistical data of the "China Tertiary Industry Statistical Yearbook" and "China Logistics Yearbook," the value-added of "transportation, storage and postal industry" has always accounted for more than 80% of the logistics industry, which largely reflects the development trend of the logistics industry. Thus, this paper follows the previous approach [27] and uses data from the transportation, warehousing, and postal industries to replace the logistics industry. Because the statistics of the transportation, warehousing, and postal industries were only collected in some regions in 2003, comprehensive consideration of data availability and comparability was adopted, and statistical data from 2004 to 2017 were selected as the research basis for this paper.

### Variable selection and processing

First, this paper uses MaxDEApro software to run network DEA model, the network DEA model can not only measure the total efficiency(tfp) of high-quality development of logistics industry, but also divide the total efficiency into two parts, namely, the production efficiency(tfp1) and service efficiency(tfp2) of high-quality development of logistics industry which are used to represent different aspects of the development of the logistics industry. In the research process of this paper, the total efficiency of high-quality logistics industry, including production efficiency and service efficiency, and in the DID model, these three efficiencies are represented by the symbol HQD. The input and output indicators for measuring production efficiency (tfp1) and service efficiency (tfp2) are shown in Table 1.

**Table 1:** Input–output indicators for total factor productivity measurement in the logistics industry.

total efficiency(*tfp*)	production efficiency(*tfp1*)	Input indicator	Capital investment
			Labor input
			Energy input
		Output indicator	Unexpected output
			Comprehensive turnover
	service efficiency(*tfp2*)	Input indicator	
			Average salary
		Output indicator	Added value in the logistics industry

#### Capital investment

Capital stock is the main indicator for measuring capital investment. Following the practice of most scholars, this paper uses perpetual inventory method to estimate the capital input of logistics industry in a strong manufacturing province. The capital stock of each period is estimated: the perpetual inventory method = the capital stock of the previous period (1-capital depreciation rate) + the new capital stock of the current period. The formula is shown in formula (9):

$$K_{it}=K_{it-1}\times (1-\delta )+I_{it}$$ (9)

where *K*_*it*_ is the capital stock in period *t*, and *K*_*it*−1_ is the capital stock in period *t*−1 in the previous period. Since 1993, the warehousing industry has been included in the statistics of new fixed assets in the transportation, warehousing, and postal industries of various provinces. To ensure consistency, this paper uses 1993 as the base period for the capital stock of the logistics industry in a strong province. The base period of the capital stock of the logistics industry in a strong province. We divide the ratio of investment in fixed assets and capital stock in the industry in that year by dividing the fixed asset investment in transportation, warehousing, and postal industries in 1993, which is a strong province in manufacturing, to obtain the manufacturing industry in 1993. Provincial logistics industry fixed assets stock and apply the GDP price index to uniformly convert the fixed asset investment amount to the price level in 1978 to eliminate the impact of price factors; *δ* is the capital depreciation rate. We refer to Liu Bingxian (2010) for the capital depreciation rate of the transportation infrastructure industry and set it to 12.1% [28]; *I*_*it*_ is the fixed asset investment amount after eliminating the influence of price factors in the *t* period.

#### Labor input

As a modern service industry that entered rapid development in the last century, the intensive level of China's logistics industry is not high, compared with other developed regions. Therefore, labor factors continue to play a significant role in the development of the industry. In this paper, the number of employed persons (at the end of the year) in transportation, warehousing, and postal urban units from 2004 to 2017 in the strong manufacturing provinces in the “China Statistical Yearbook” is selected as the input variable of labor factors in the logistics industry.

#### Energy input

China's logistics industry remains in the initial stage of development based on traditional transportation. Therefore, various types of energy, especially refined oil, are important input factors for the development of the logistics industry. Due to the different units of measurement for different types of energy, we adopt the descaling process, such as formula (10):

$$E_{i}=\sum_{j=1}^{12}\left(X_{j}\times P_{j})(i=1,2\ldots 10\right)$$ (10)

Among them, *E*_*i*_ is the total energy consumption of each manufacturing province (unit: 10,000 tons of standard coal), and *X*_*j*_ is the 12 types of energy commonly used in the transportation, storage, and postal industries: raw coal, washed coal, coke, coke furnace gas, crude oil, gasoline, kerosene, diesel, fuel oil, liquefied petroleum gas, refinery dry gas, natural gas, heat, and electricity. *P*_*j*_ is the standard coal coefficient for various types of energy in the "GB/T 2589–2008 General Calculation of Comprehensive Energy Consumption."

#### Carbon emissions output

Under environmental constraints, pollution emissions are the most important undesired outputs that accompany the expected output during the development of the logistics industry and mainly include carbon dioxide, methane, and nitrous oxide, of which carbon dioxide is the largest and most representative of the pollution emissions; therefore, we use *CO*_2_ emissions from the logistics industry in a strong manufacturing province as a variable of undesired output. There is no authoritative statistical data on the value of carbon emissions. This paper refers to the literature and uses the formulas in the "Guide to the Calculation Tool for Greenhouse Gas Emissions Caused by Energy Consumption (Version 2.1)," based on the relevant data of each energy consumption, to specifically measure each manufacturing the carbon emission value of the logistics industry in a strong province. The formula is shown in formula (11):

$$CO_{2}=\sum_{j=1}^{10}M_{j}\times HV_{j}\times OX_{j}\times C_{j}\times (\frac{44}{12})\times 10^{-6}$$ (11)

where *M*_*j*_ is 10 types of direct burning mineral energy, *HV*_*j*_ is the caloric value of energy *j* based on weight or volume, *OX*_*j*_ is the oxidation rate of energy *j* during combustion, and *C* is the carbon content value of energy *j* based on the calorific value. Two types of indirect energy—electricity and heat—are calculated by multiplying the power and heat emission factors for each year shown in the “Guide (Version 2.1)” and based on the consumption.

#### Comprehensive turnover

The turnover of the logistics industry mainly considers three transportation modes: railway, waterway, and highway, and converts the passenger turnover and cargo turnover into comprehensive turnover.

#### Value-added output of logistics industry

The added value of the logistics industry refers to the sum of the value created by the logistics industry within a certain period of time; it is the most core and key indicator that reflects the expected output of the logistics industry. In this paper, the value-added output value of the logistics industry in the strong manufacturing industry uses the value-added of transportation, warehousing, and postal services. The data are from the “China Statistical Yearbook” (2004–2017), and the GDP deflator (1978 as the base year) is used to process the added value data and eliminate the influence of price factors.

Second, the index system of the explanatory variables and the explained variables in the regression equation are shown in Table 2.

**Table 2:** Index system of explanatory variables and explained variables in regression equations.

• Explained variable	Production/Service/Total efficiency	*HQD(tfp1/tfp2/tfp)*	Network DEA calculation
Core explanatory variables	Regional grouping	*id*	Provinces with pilot areas take 1, otherwise 0
	Time grouping	*time*	Take 1 after 2015, otherwise take 0
Explanatory variables	Governmental support	*GI*	Proportion of fiscal expenditure of logistics industry in local fiscal expenditure to measure the government's support for the logistics industry
	Scale of economic growth	*GDP*	National GDP as an indicator of the scale of economic growth
	Investment in fixed assets	*FAI*	Taking the investment amount of the fixed assets of the whole society as an index to evaluate the productivity of the logistics industry
	Retail sales of consumer goods	*SCGS*	Taking the retail sales of social consumer goods as a critical indicator to examine the impact mechanism of logistics industry productivity
	Industrial structure	*FIVA/SIVA/TIVA*	Take the added value of the primary industry, the secondary industry, and the tertiary industry as indicators to measure the state of industrial structure adjustment
	Average salary	*AW*	Taking the average wage of employees in the logistics industry as one of the indicators to examine the production/service efficiency of the logistics industry

### Data descriptive statistics

Based on the aforementioned data collection and processing methods, a descriptive statistical analysis is performed on the explanatory variables and the interpreted variables from 2004 to 2017 (Using stata14 software). The statistical information of each element is shown in Table 3.

**Table 3:** Descriptive statistics of each variable.

Variable	Mean value	Standard deviation	Minimum value	Median	Maximum value
*tfp1*	0.157	0.0542	0.068	0.149	0.321
*tfp2*	0.312	0.2104	0.068	0.242	1.000
*gdp*	2.564	1.9818	0.305	1.931	8.971
*scgs*	0.984	0.8301	0.086	0.713	3.820
*fai*	1.349	1.2702	0.003	0.913	5.520
*fiva*	0.156	0.1373	0.008	0.129	0.498
*siva*	1.180	0.9485	0.139	0.820	3.866
*tiva*	1.216	0.9808	0.123	0.904	4.749
*gi*	0.043	0.0293	0.001	0.043	0.217
*aw*	4.518	2.653	0.6273	4.429	11.676

According to Table 3, the minimum values of production efficiency and service efficiency measured by the network DEA representing the high-quality development of the logistics industry are 0.068 and 0.068, the maximum values are 0.321 and 1, respectively, and the average values are 0.157 and 0.312. This finding indicates a large difference between the two efficiencies during the sample period, and a large difference between the two efficiency values. The other control variables have similar situations on the basis of the statistical table, which provide an empirical basis for the test results of the DID model described later.

## Empirical analysis

To investigate whether and how the policy of the "Made in China 2025" strategy affects the production efficiency and service efficiency of the logistics industry, this paper conducts the following three empirical tests in the following order: We draw the trend chart of the production efficiency and service efficiency of the Study group and the Reference group and then observe the change trend; use the single variable DID to preliminarily investigate the impact of the "Made in China 2025" strategic policy on the production efficiency and service efficiency of the logistics industry; introduce the control variables; and use the DID model with time fixed effect for the empirical test.

### Time trend of production efficiency and service efficiency in the logistics industry

**Fig 2:**
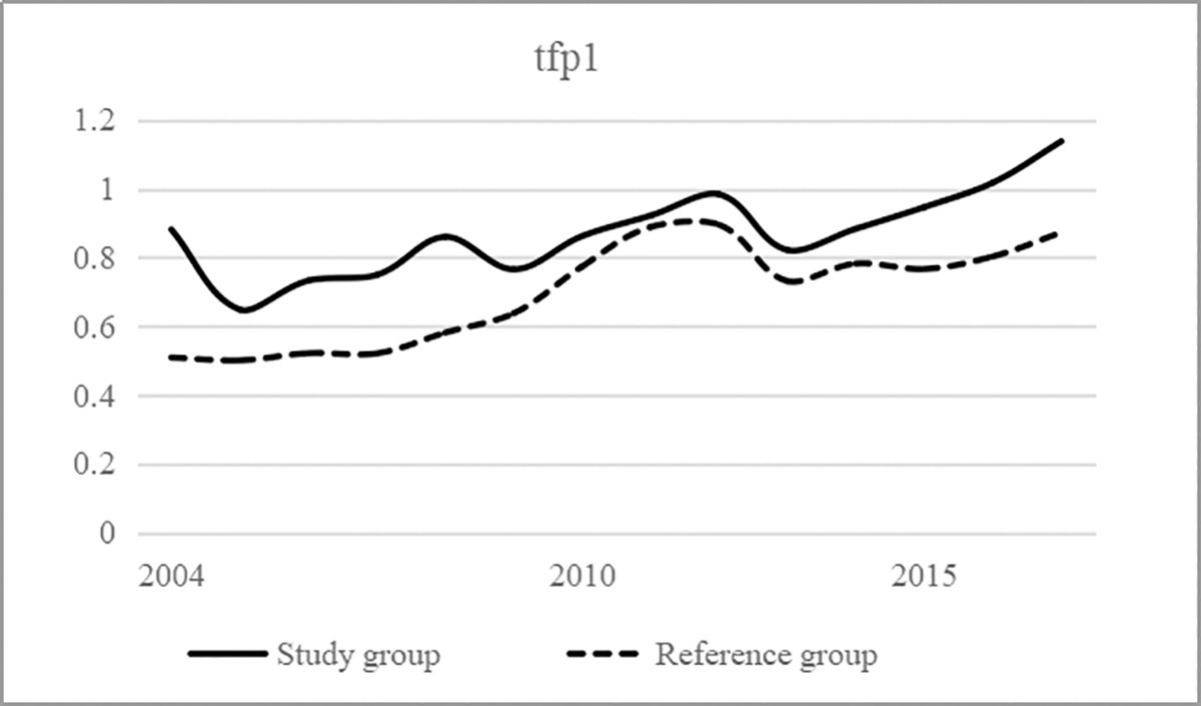
Changes in production efficiency of the logistics industry.

**Fig 3:**
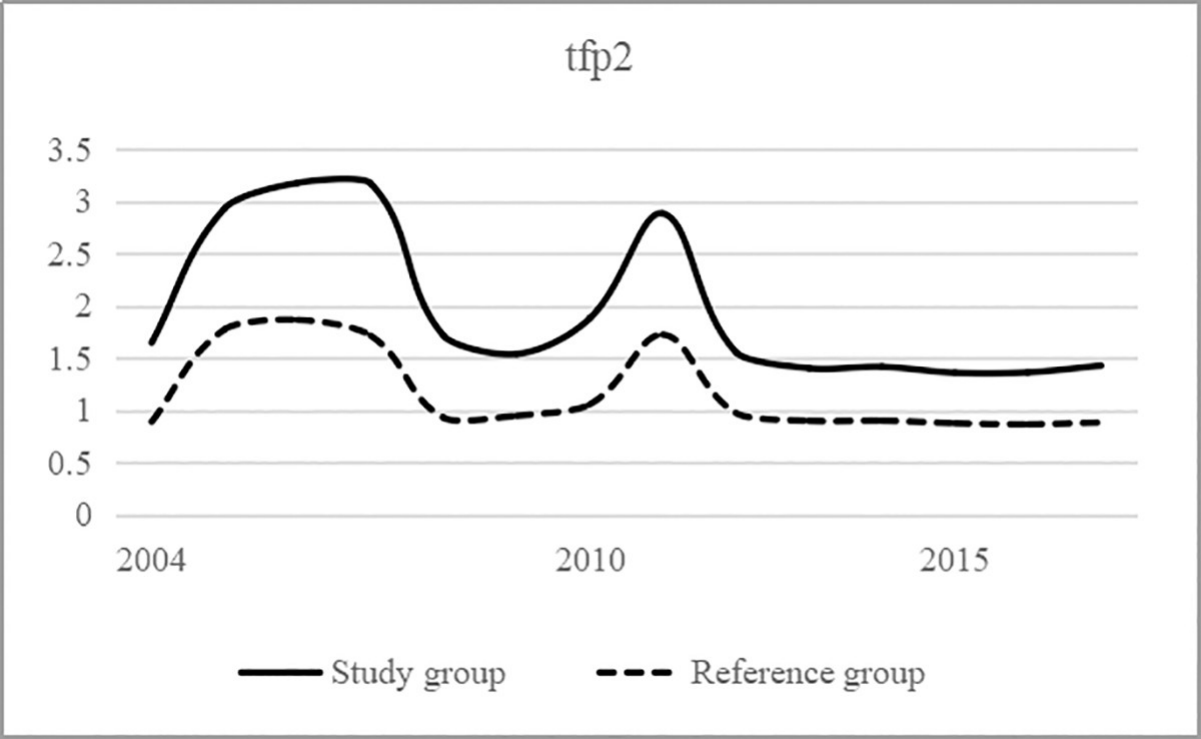
Changes in service efficiency of the logistics industry.

Based on calculating the production efficiency and service efficiency of the logistics industry, this paper draws the time trend chart of these factors for the Study group and Reference group in Figs 2 and 3, in which their time changes and differences are intuitively shown. Before the "manufacturing industry development" strategy was introduced, there was no regular change in production efficiency, but after the strategy was introduced, the gap between the Study group and the Reference group significantly widened. However, in sharp contrast, the service efficiency of the Study group and Reference group before the strategy was introduced basically maintained a parallel time trend, and the gap between the two groups slightly widened after the strategy was introduced, but the difference was not obvious.

### Univariate analysis

First, before using the DID method to test the effect of "manufacturing industry development" strategies, this paper conducts univariate test on the agent variable production efficiency and service efficiency of the high-quality development of the logistics industry. The Study group and Reference group are the same as the settings in the previous DID model. The inspection results are shown in Table 4. We observe that before the implementation of the "manufacturing development" strategy, the production efficiency of the Study group is 0.023 higher than that of the Study group at a significant level of 5%, and the service efficiency of the Study group is 0.0631 lower than that of the Reference group but not significantly. After the implementation of the "manufacturing development" strategy, the production efficiency of the Study group is 0.039 higher than that of the Study group at a significant level of 5%, which indicates that the implementation of the "manufacturing development" strategy has positively enlarged the high-quality development level represented by the production efficiency between the Study group and the Reference group, with remarkable results. However, after the implementation of the strategy, the service efficiency has negatively enlarged the high-quality development level represented by the service efficiency of the Study group and the Reference group, which could indicates that in the short term, the implementation of the strategy may have a restraining effect on the logistics service, but the specific statistical results and significance of these two differences in the DID model must be further explored.

**Table 4:** "manufacturing development" strategy and high-quality development of China's logistics industry: Single variable t-test results.

		Study group(1)	Reference group(2)	Difference (1)-(2)	T-test (1)-(2)
*tfp1*	Before the policy	0.167 (0.006)	0.144 (0.007)	0.023 (0.010)	2.339***
	After the policy	0.183 (0.015)	0.144 (0.015)	0.039 (0.021)	1.833***
*tfp2*	Before the policy	0.279 (0.025)	0.342 (0.306)	-0.063 (0.039)	-1.610
	After the policy	0.263 (0.047)	0.376 (0.661)	-0.113 (0.813)	-1.391

***p<0.05,standard error in brackets, same below.

### Estimation results

According to the univariate analysis of this paper, we observe that the "manufacturing development" strategy has positively enlarged the gap in the production efficiency between the Study group and the Reference group but negatively enlarged the gap of the service efficiency between the Study group and the Reference group. Thus, we cannot draw a conclusion on the "manufacturing development" strategy and the high-quality development of the logistics industry. Statistical significance analysis that controls other influencing factors is also necessary. Based on the original DID model, this paper further controls the individual effect, time effect, and individual time trend, and then estimates and analyzes after adding the industry level control variables. The results are shown in Table 5, columns (1) and (2).

**Table 5:** Estimation results based on the DID model.

	Average effect		Dynamic effect	
	*tfp1*(1)	*tfp2*(2)	*tfp1*(3)	*tfp2*(4)
id*time	0.499*** (0.0000)	1.588*** (0.0001)		
treat*year2015			0.254*** (0.0000)	0.257*** (0.0001)
treat*year2016			0.904*** (0.0004)	0.167*** (0.0018)
treat*year2017			1.586*** (0.0012)	-2.230*** (0.0053)
*gdp*	-2.220*** (0.0002)	-9.882*** (0.0004)	3.478*** (0.0071)	-29.891*** (0.0313)
*Scgs*	0.963*** (0.0003)	-2.8420** (0.0006)	-2.558*** (0.0035)	9.519** (0.0157)
*Fai*	-0.727*** (0.0002)	-0.5537*** (0.0003)	-1.413*** (0.0011)	1.856*** (0.0048)
*Fiva*	5.820*** (0.0047)	-11.223** (0.0088)	7.466*** (0.0101)	-17.004*** (0.0447)
*Siva*	2.318** (0.0000)	7.989*** (0.0001)	0.357*** (0.0023)	14.874*** (0.0101)
*Tiva*	1.253*** (0.0004)	11.233** (0.0008)	-3.873** (0.0069)	29.234*** (0.0304)
*Gi*	-2.473*** (0.0011)	-4.775*** (0.0020)	19.986*** (0.0280)	-83.644*** (0.1240)
*Aw*	-0.479*** (0.0000)	-1.885*** (0.0001)	-0.317*** (0.0002)	-2.453** (0.0009)
*Cons*	2.141*** (0.0006)	10.689*** (0.0010)	0.813*** (0.0023)	15.353*** (0.0102)
*Year*	yes	yes	yes	yes
*Firm*	yes	yes	yes	yes
id*Year	yes	yes	yes	yes
*N*	140	140	140	140
*R*^*2*^	0.659	0.304	0.540	0.297

According to the regression results in Table 5, the coefficients of the cross terms that represent the implementation effect of policies are positive in production efficiency and service efficiency and are highly significant at a significant level of 5%, which fully shows that "manufacturing development" significantly improves the production efficiency and service efficiency of the logistics industry, that is, "manufacturing development" promotes the high-quality development of the logistics industry. However, based on columns (1) and (2) of Table 5, we cannot explain why the gap of service efficiency between the Study group and the Reference group was negatively enlarged because of the "manufacturing development" strategy. Therefore, to conduct an estimation from the perspective of dynamic effect, this paper further explores the policy effect and change trend of "manufacturing industry development" strategy year by year using model (2).

The results are shown in columns (3) and (4) of Table 5. We observe in Table 5 that when the production efficiency is regressed, the influence coefficient of "manufacturing development" strategy on the first year (2015) is 0.254, the second year (2016) is 0.904, and the third year (2017) is 1.586, and the estimation results are highly significant at the level of 5%. We observe that that the boosting effect of the "manufacturing industry development" strategy on production efficiency is increasing by year, which shows that the implementation of a "manufacturing industry development" strategy requires a certain period of time and still requires time to promote the steady development of the logistics industry in a high-quality manner by adhering to the basic policy of "innovation driven, quality first, green development, structural optimization and talent oriented."

We observe that based on column (4) of Table 5, the coefficient of the "manufacturing industry development" strategy on the high-quality development of the logistics industry represented by service efficiency in the first year (2015) is 0.257, in the second year (2016) is 0.167, and in the third year (2017) is -2.230, and the estimated results are highly significant at the level of 5%. This finding shows that the "manufacturing development" strategy does not show a trend of increasing by year for service efficiency, and the boosting effect in 2017 is significantly negative, which is consistent with the results of the aforementioned univariate analysis.

The logistics industry is a productive service industry that provides value-added services for the production and services of the first, second, and third industries. After the strategy of "manufacturing development" was proposed, many resources were allocated to the development of the manufacturing industry, which occupied the resources of other industries. Additionally, the service industry in the logistics industry was mainly provided for the tourism industry. The resources of a region are limited in a given period of time. Therefore, under the strategic guidance of "manufacturing industry development," resources flow more to the production department, resulting in the influence coefficient of the strategy on service process efficiency decreasing year by year.

In addition, in response to the policy of "green development" and "quality first" in high-quality development, this paper considers the unexpected output when calculating its production efficiency. In recent years, the centralized outbreak of logistics technology has promoted the development of intelligent equipment manufacturing and logistics robots and pushed logistics into the mature stage of equipment standardization and load unitization. However, service efficiency more reflects "talent-oriented" policy. At the beginning of the "manufacturing development" strategy, the policy dividend from the strategy is large, which has a significant effect on promoting the service efficiency of the logistics industry. However, with the passage of time, the dividend gradually disappears, which changes the original means to promote the service efficiency of the logistics industry. In this manner, the promotion of policy to high-quality development represented by service efficiency can no longer be continued, that is, the influence coefficient of this strategy on service process efficiency may decrease by year.

In this case, we should accelerate transformation through technological progress and the improvement of productivity and the quality of labor in the industry, to realize the long-term growth. The regression results of other control variables are significant, indicating that they have a good fitting effect and control effect on the model, and the control variables selected in the following are also shown at a significant level. Limited by space, we present the regression results of the control variables in Table 5.

### How does the "manufacturing development" strategy promote the high-quality development of China's logistics industry: From the perspective of the technological innovation mode

Based on the aforementioned study, the "manufacturing development" strategy promotes the overall high-quality development of the logistics industry and makes substantial contributions to the structural upgrading and role transformation of China's manufacturing industry, as well as the low-carbon transformation in China. Additionally, this paper holds that technological innovation is the main driver that promotes the high-quality development of China's logistics industry. Because there are differences in resource endowments and technological demands in different industries, this paper divides the technological innovation index into four categories according to the mode of technological innovation: independent innovation, imitative innovation, direct innovation and indirect innovation). Combined with the index selection in the literature [29, 30], this paper uses "R&D investment intensity" to measure independent innovation (innovin), uses "above scale industrial enterprises digest and absorb technology expenditure" to measure imitative innovation (innovim), uses "number of foreign technology import contracts " to measure direct import (innovdi), and uses "actual use of foreign direct investment" to measure indirect import (innovii). We establish the following model (12):

$$\begin{array}{l} HQD_{it}=\theta_{1}(id_{it}\times time_{it}\times innovin_{it})+\theta_{2}(id_{it}\times time_{it})+\theta_{3}innovin+\\ \;{{}_{}}_{}{{}_{}}_{}{{}_{}}_{}{{}_{}}_{}{{}_{}}_{}{{}_{}}_{}\alpha x_{it}+\lambda_{t}+\mu_{i}+yeartrend_{i}+\varepsilon_{it} \end{array}$$ (12)

Among them, *innovin*_*it*_ represents independent innovation, and the other variables are the same as aforementioned. In addition, the model including imitation innovation, direct innovation, or indirect innovation is the same as model (12).

From the perspective of technological innovation, the regression results of the "manufacturing development" strategy's boosting effect on the high-quality development of the logistics industry are shown in Tables 6 and 7

**Table 6:** Impact of the "manufacturing development" strategy on the high-quality development of the logistics industry from the perspective of technological innovation (production efficiency).

	*tfp1*(1)	*tfp1*(2)	*tfp1*(3)	*tfp1*(4)
id*time	1.132*** (0.0004)	0.359*** (2.34e-06)	0.822*** (0.0021)	0.801*** (0.0000)
*innovin*	0.146*** (0.0001)			
Id*time *innovin	0.770*** (0.0002)			
*innovim*		-0.020*** (4.97e-06)		
Id*time *innovim		0.301*** (2.70e-06)		
*innovdi*			1.084*** (0.0067)	
id*time *innovdi			-4.794*** (0.0290)	
*innovii*				-0.0002*** (0.0000)
id*time *innovii				-0.003*** (0.0000)
*cons*	2.310*** (0.0004)	1.823*** (0.0002)	9.582*** (0.0482)	1.897*** (0.0003)
control variables	yes	yes	yes	yes
*Year*	yes	yes	yes	yes
*Firm*	yes	yes	yes	yes
id*Year	yes	yes	yes	yes
*N*	140	140	140	140
*R*^*2*^	0.208	0.182	0.288	0.130

**Table 7:** Impact of "manufacturing development" on the high-quality development of the logistics industry from the perspective of technological innovation (service efficiency).

	*tfp2*	*tfp2*	*tfp2*	*tfp2*
id*time	-0.733*** (0.0003)	1.044*** (0.0270)	2.032*** (0.0011)	1.415*** (0.0000)
*innovin*	4.992*** (0.0004)			
id*time *innovin	1.220*** (0.0003)			
*innovim*		0.035*** (0.0007)		
id*time *innovim		-0.120*** (0.0086)		
*innovdi*			0.912*** (0.0035)	
id*time *innovdi			-6.700*** (0.0149)	
*innovii*				0.004*** (0.0000)
id*time *innovii				-0.009** (0.0000)
*cons*	9.491*** (0.0007)	9.827*** (0.1962)	13.891*** (0.0248)	1.897*** (0.0004)
control variables	yes	yes	yes	yes
*Year*	yes	yes	yes	yes
*Firm*	yes	yes	yes	yes
id*Year	yes	yes	yes	yes
*N*	140	140	140	140
*R*^*2*^	0.514	0.427	0.427	0.499

According to the regression results in Tables 6 and 7, under the influence of the "manufacturing development" strategy, different technological innovations have different effects on boosting the high-quality development of China's logistics industry. Independent innovation has a significant positive role in promoting the high-quality development of the logistics industry represented by production efficiency, with a coefficient of 0.770. Technological innovation in other forms does not have a significant positive effect. Similarly, when researching service efficiency, only independent innovation has a significant positive effect, with a coefficient of 2.388. This result shows that at present, China's logistics industry is in a period of large demand for logistics equipment, and multilevel demand coexists. Thus, China should focus on improving its R&D innovation level, which could greatly improve the high-quality development level of the logistics industry. In addition to the path of independent innovation, the other three means of technological innovation have no significant effect on improving the high-quality development of China's logistics industry in the short term. In conclusion, we confirm that the strategy of "manufacturing development" can boost the high-quality development of China 's logistics industry, and from the perspective of technological innovation, independent innovation is the main boost path.

In this paper, the method of substitution of surrogate variables is used to test the robustness of the regression. The index representing the high-quality development of the logistics industry is replaced by the total efficiency value, and then the DID model is used for regression analysis. The results are shown in Table 8 and Table 9. The results of the robustness test are similar to the previous test and indicate that the relevant conclusions of this paper are correct and trustworthy.

**Table 8:** Robustness test results.

	Average effect	Dynamic effect
	*tfp*(1)	*tfp*(2)
id*time	1.043*** (0.0001)	
treat*year2015		0.255*** (0.0000)
treat*year2016		0.535*** (0.0012)
treat*year2017		-0.322*** (0.0036)
*gdp*	-6.051*** (0.0002)	-13.207*** (0.0210)
*scgs*	-0.940*** (0.0003)	3.481*** (0.0105)
*fai*	-0.640*** (0.0002)	0.221*** (0.0032)
*fiva*	-2.701*** (0.0053)	-4.768*** (0.0300)
*siva*	5.153*** (0.0000)	7.616*** (0.0068)
*tiva*	6.2431*** 0.0005)	12.681*** (0.0204)
*gi*	-3.624*** (0.0012)	-31.829*** (0.0833)
*aw*	-1.182*** (0.0000)	-1.385*** (0.0006)
*cons*	6.415*** (0.0006)	8.083*** (0.0069)
*Year*	yes	yes
*Firm*	yes	yes
id*Year	yes	yes
*N*	140	140
*R*^*2*^	0.876	0.943

**Table 9:** Robustness test results from the perspective of technological innovation.

	*tfp*	*tfp*	*tfp*	*tfp*
id*time	-2.862*** (0.0004)	0.580*** (0.0000)	1.427*** (0.0013)	1.108*** (0.0000)
*innovin*	1.019*** (0.0001)			
id*time *innovin	1.579*** (0.0002)			
*innovim*		0.0793*** (0.0000)		
id*time *innovim		0.5303*** (0.0000)		
*innovdi*			0.998*** (0.0041)	
id*time *innovdi			-5.747*** (0.0176)	
*innovii*				0.002*** (0.0000)
id*time *innovii				-0.006*** (0.0000)
*cons*	5.490*** (0.0005)	-0.679 (0.0002)	11.737*** (0.0293)	4.380*** (0.0002)
control variables	yes	yes	yes	yes
*Year*	yes	yes	yes	yes
*Firm*	yes	yes	yes	yes
id*Year	yes	yes	yes	yes
*N*	140	140	140	140
*R*^*2*^	0.421	0.587	0.361	0.440

When using DID model to evaluate the effect of policy, the ideal situation is that the individual characteristics of the study group and the reference group should be the same. If this condition cannot be met,it may cause “selective bias”. To avoid this problem, this paper further uses the Propensity Score Matching (PSM) to re-estimate by matching the reference group, to ensure the robustness of the results. The results are shown in Table 10, it’s similar to the previous results. It means the conclusions of this paper are correct and trustworthy.

**Table 10:** Robustness test results: Proportioning reference group by PSM method.

	*tfp*1(1)	*tfp*2(2)
id*time	0.287*** (0.0003)	0.694*** (0.0000)
*gdp*	3.572*** (0.0030)	-8.344*** (0.0004)
*scgs*	4.395*** (0.0026)	4.362*** (0.0002)
*fai*	-2.030*** (0.0012)	0.845*** (0.0000)
*fiva*	46.299*** (0.0357)	33.153*** (0.0012)
*siva*	-2.855*** (0.0022)	8.378*** (0.0002)
*tiva*	-4.601*** (0.0037)	4.601*** (0.0006)
*gi*	23.857*** (0.0166)	56.154*** (0.0006)
*aw*	-0.473*** (0.0003)	-0.600*** (0.0000)
*cons*	-4.497*** (0.0038)	-1.174*** (0.0000)
*Year*	Yes	Yes
*Firm*	Yes	Yes
id*Year	Yes	Yes
*N*	115	115
*R*^*2*^	0.884	0.965

## Main conclusions and policy suggestions

### Main conclusions

#### The strategy of "manufacturing development" has a significant positive impact on the high-quality development of the logistics industry

According to the regression results in Table 5, the coefficient of the cross term (*id*_it_*time_*it*_) representing the effect of policy implementation is positive in both production efficiency and service efficiency, and it is highly significant at a significant level of 5%, which fully shows that the "manufacturing development" has significantly improved the production efficiency and service efficiency of the logistics industry, that is, the "manufacturing development" has promoted the high-quality development of the logistics industry.

#### The influence coefficient of the "manufacturing development" strategy on the logistics production process increases annually, and that on the service process decreases annually

In the regression of production efficiency, the influence coefficient of the "manufacturing development" strategy on the first year (2015) of high-quality development represented by production efficiency is 0.254, the second year (2016) is 0.904, and the third year (2017) is 1.586, and the estimated results are highly significant at a significant level of 5%. We observe that the promotion effect of the "manufacturing development" strategy on production efficiency is increasing by year, which shows that the implementation of the "manufacturing development" strategy requires a certain period of time, and its basic policy of "innovation driven, quality first, green development, structural optimization, and talent oriented" still requires a long time to steadily penetrate into the development of the logistics industry. We observe from column (4) of Table 5 that the influence coefficient of the "manufacturing development" strategy on the first year (2015) of high-quality development of the logistics industry represented by service efficiency is 0.257, the second year (2016) is 0.167, and the third year (2017) is—2.230, and the estimated results are highly significant at the level of 5%. This finding shows that the "manufacturing development" strategy does not make the service efficiency increase by year, and the effect in 2017 is significantly negative.

#### Independent innovation is the main means for "manufacturing development" to boost the high-quality development of the logistics industry

Under the promotion of the "manufacturing development" strategy, the effect of technological innovation from different sources on boosting the high-quality development of China's logistics industry differs. Independent innovation plays a significant positive role in promoting the high-quality development of the logistics industry represented by production efficiency, with a coefficient of 0.770. Technological innovation from other sources does not have a significant positive effect. Independent innovation plays a significant positive role in promoting the high-quality development of the logistics industry represented by production efficiency, with a coefficient of 0.770. Technological innovation from other sources does not have a significant positive effect. Similarly, in the research on the high-quality development of the logistics industry represented by service efficiency, only independent innovation has a significant positive effect, with a coefficient of 2.388. In addition to independent innovation, the other three sources of technological innovation have no significant effect on improving the high-quality development of China's logistics industry in the short term.

### Policy suggestions

1. Strengthen the coordination and interaction between the two industries to achieve high-quality joint development. The basic characteristics of the joint development of the manufacturing industry and logistics industry require that the logistics industry and manufacturing industry can achieve collaborative and mutual progress in all stages of economic development. As the leading region of China's manufacturing industry development, strong manufacturing provinces have begun to move towards the stage of new development, whereas the logistics industry remains in the early stage of scale expansion. In the context of high-quality development, it is necessary to actively adjust the strategic objectives of high-quality development of the logistics industry, according to the needs of the development of the manufacturing industry; properly manage the linkage between energy conservation and emissions reduction, manufacturing production, and logistics operation; and realize the efficient, high-quality coordinated development of the two industries.
2. Understand the opportunities created by the strategy of "manufacturing development." Relying on the development of advanced manufacturing industry, China must improve the technical level of its logistics industry. The "manufacturing development" strategy does not promote an annual increase in service efficiency, and the promotion effect in 2017 is significantly negative. Therefore, improving independent innovation is a critical means to achieve the high-quality development of the logistics industry in a province with a strong manufacturing industry. It is necessary to give full play to the advanced manufacturing capacity of a strong manufacturing province; improve and perfect the functions of the logistics industry; actively develop modern energy and emission reduction technology; update the facilities and equipment in various links of the logistics industry, such as packaging, transportation, and inventory; and adopt modern information technology to optimize the application environment of the logistics industry technology, which can largely help to improve the production efficiency and service efficiency of logistics, and promote the transformation of logistics industry from scale-oriented to technology-oriented.
3. Adhere to innovation-driven development strategy and rational allocation of logistics industry input elements. During the inspection period, from the perspective of technological innovation, only independent innovation is the main path of the "manufacturing development" strategy to promote the high-quality development of the logistics industry in China, and the other innovative means have not been advantageous, which greatly restricts the promotion of high-quality development of the logistics industry. Therefore, China must objectively assess the scale and level of logistics development in each strong manufacturing province, adjust the input quantity and proportion of all types of resource elements in the logistics industry in time, and force logistics enterprises to improve the utilization efficiency of input elements. According to the development stage of the logistics industry, each strong manufacturing province should integrate the innovation concept into the development strategy of the logistics industry, formulate the high-quality development plan according to the local conditions, scientifically and reasonably set the development objectives of each stage, and orderly promote the comprehensive development of the logistics industry of the strong manufacturing province.

## Discussion, limitation, and future work

To some extent, this paper supplements the shortage of research about impacts of manufacturing policies on the logistics industry, and empirically studies that manufacturing policy has a significantly positive impact on the logistics industry high-quality development, and independent innovation is the main path to promote the effects of the high-quality development of logistics industry.

However, this paper has some limitations in data selection and classification. The manufacturing policy based on "Made in China 2025" may be slightly macro. At the same time, due to the availability of data, this paper takes the provincial area as the research object, which makes the amount of data relatively limited.

In the subsequent research, the research group will sort out and classify the manufacturing policies, and further explore the effects of policy synergy on the efficiency of logistics industry. This paper will continue to search for prefecture-level city data to expand sample size.

## Supporting information

S1 Table The data of all variables. (DOCX)
