# Correction: Has the manufacturing policy helped to promote the logistics industry?

**DOI:** 10.1371/journal.pone.0268208

**Published:** 2022-05-03

**Authors:** Dan He, Jialiang Yang, Zhengming Wang, Wenchao Li

The following information is missing from the Funding statement: This study received funding from the Jiangsu Social Science Fund (18EYC006; http://jspopss.jschina.com.cn) and the National Natural Science Foundation of China (71704067/71974078; http://www.nsfc.gov.cn), both awarded to DH. The funders had no role in study design, data collection and analysis, decision to publish, or preparation of the manuscript.

## References

[1] He D, Yang J, Wang Z, Li W (2020) Has the manufacturing policy helped to promote the logistics industry? PLoS ONE 15(7): e0235292. 10.1371/journal.pone.0235292 32614879 PMC7332018

