# Supplementary material for: Has the manufacturing policy helped to promote the logistics industry?
Source: PLoS One. 2020 Jul 2;15(7):e0235292. doi: 10.1371/journal.pone.0235292 (PMC7332018; doi:10.1371/journal.pone.0235292)
Supplement: S1 Table — (DOCX) [file pone.0235292.s001.docx]

| id | year | tfp | tfp1 | tfp2 | tp | post | treat | ty15 | ty16 | ty17 | GDP | SCGS | FAI | FIVA | SIVA | TIVA | GI | AW | innov-YS | innov-MF | innov-ZJYJ | innov-JJYJ |
| --- | --- | --- | --- | --- | --- | --- | --- | --- | --- | --- | --- | --- | --- | --- | --- | --- | --- | --- | --- | --- | --- | --- |
| BJ | 2004 | 0.147995 | 0.142296 | 0.153694 | 0 | 0 | 0 | 0 | 0 | 0 | 0.60332 | 0.26266 | 0.25283 | 0.00853 | 0.18455 | 0.41024 | 0.006164 | 2.3909 | 5.24 | 0.3607 | 1.615 | 30.8354 |
| BJ | 2005 | 0.09609 | 0.102963 | 0.089217 | 0 | 0 | 0 | 0 | 0 | 0 | 0.696952 | 0.29117 | 0.00841 | 0.008868 | 0.202651 | 0.485433 | 0.006114 | 1.4677 | 5.55 | 0.0455 | 2.013 | 35.2638 |
| BJ | 2006 | 0.137221 | 0.082666 | 0.191775 | 0 | 0 | 0 | 0 | 0 | 0 | 0.81178 | 0.32953 | 0.33715 | 0.00854 | 0.21779 | 0.58545 | 0.005704 | 1.5235 | 5.33 | 1.1441 | 1.857 | 45.5191 |
| BJ | 2007 | 0.206675 | 0.093569 | 0.31978 | 0 | 0 | 0 | 0 | 0 | 0 | 0.98468 | 0.38352 | 0.39666 | 0.00994 | 0.24939 | 0.72535 | 0.016004 | 1.7012 | 5.13 | 1.6143 | 1.711 | 50.6572 |
| BJ | 2008 | 0.194201 | 0.208866 | 0.179537 | 0 | 0 | 0 | 0 | 0 | 0 | 1.1115 | 0.46455 | 0.38485 | 0.01114 | 0.25929 | 0.84107 | 0.033466 | 4.6043 | 4.95 | 2.455 | 1.56 | 60.8172 |
| BJ | 2009 | 0.226894 | 0.164494 | 0.289294 | 0 | 0 | 0 | 0 | 0 | 0 | 1.2153 | 0.53099 | 0.41496 | 0.01168 | 0.28042 | 0.9232 | 0.052137 | 4.6087 | 5.5 | 1.1873 | 1.275 | 61.2094 |
| BJ | 2010 | 0.216819 | 0.191623 | 0.242015 | 0 | 0 | 0 | 0 | 0 | 0 | 1.37779 | 0.62293 | 0.54935 | 0.01243 | 0.33231 | 1.03305 | 0.038128 | 5.5912 | 5.82 | 0.72744 | 1.359 | 63.6358 |
| BJ | 2011 | 0.295079 | 0.158728 | 0.43143 | 0 | 0 | 0 | 0 | 0 | 0 | 1.60004 | 0.69003 | 0.59106 | 0.01362 | 0.37444 | 1.21198 | 0.043524 | 2.5293 | 5.76 | 7.0576 | 1.388 | 70.5447 |
| BJ | 2012 | 0.330652 | 0.157763 | 0.50354 | 0 | 0 | 0 | 0 | 0 | 0 | 1.7801 | 0.77028 | 0.64628 | 0.01503 | 0.40583 | 1.35924 | 0.066144 | 6.5986 | 5.95 | 4.3852 | 1.343 | 80.416 |
| BJ | 2013 | 0.110957 | 0.088046 | 0.133869 | 0 | 0 | 0 | 0 | 0 | 0 | 1.98008 | 0.88721 | 0.70322 | 0.01596 | 0.42926 | 1.53486 | 0.055536 | 7.2006 | 5.98 | 5.69934 | 1.067 | 85.2418 |
| BJ | 2014 | 0.193227 | 0.103455 | 0.282999 | 0 | 0 | 0 | 0 | 0 | 0 | 2.13308 | 0.9638 | 0.75623 | 0.0159 | 0.45455 | 1.66263 | 0.047418 | 7.8183 | 5.95 | 7.6581 | 0.859 | 90.4085 |
| BJ | 2015 | 0.128462 | 0.096544 | 0.160379 | 0 | 1 | 0 | 0 | 0 | 0 | 2.301459 | 1.0338 | 0.79909 | 0.01402 | 0.45426 | 1.83317 | 0.051524 | 8.1695 | 6.01 | 7.5201 | 0.634 | 129.9635 |
| BJ | 2016 | 0.263306 | 0.089536 | 0.437077 | 0 | 1 | 0 | 0 | 0 | 0 | 2.5669 | 1.1005 | 0.78887 | 0.01296 | 0.47744 | 1.99953 | 0.055173 | 9.0682 | 5.96 | 7.18254 | 0.543 | 130.2858 |
| BJ | 2017 | 0.405873 | 0.1195 | 0.692246 | 0 | 1 | 0 | 0 | 0 | 0 | 2.801494 | 1.15754 | 0.83704 | 0.01204 | 0.53268 | 2.25678 | 0.065423 | 9.7567 | 5.755956 | 7.55283 | 1.161096 | 120.1512 |
| TJ | 2004 | 0.185447 | 0.111175 | 0.259719 | 0 | 0 | 0 | 0 | 0 | 0 | 0.314135 | 0.104478 | 0.125898 | 0.010528 | 0.170802 | 0.132805 | 0.006193 | 2.5723 | 1.73 | 0.9027 | 0.489 | 24.7243 |
| TJ | 2005 | 0.376538 | 0.164163 | 0.588912 | 0 | 0 | 0 | 0 | 0 | 0 | 0.394794 | 0.12016 | 0.005804 | 0.011238 | 0.216583 | 0.166973 | 0.006034 | 1.459 | 1.96 | 16.6667 | 0.509 | 33.2885 |
| TJ | 2006 | 0.265817 | 0.102144 | 0.42949 | 0 | 0 | 0 | 0 | 0 | 0 | 0.451894 | 0.13831 | 0.18498 | 0.010335 | 0.249792 | 0.191767 | 0.005643 | 1.7463 | 2.13 | 3.9212 | 0.55 | 41.3077 |
| TJ | 2007 | 0.555435 | 0.162851 | 0.94802 | 0 | 0 | 0 | 0 | 0 | 0 | 0.532068 | 0.16506 | 0.238863 | 0.011019 | 0.294183 | 0.226866 | 0.012591 | 1.8781 | 2.18 | 3.5431 | 0.475 | 52.7776 |
| TJ | 2008 | 0.40773 | 0.108634 | 0.706826 | 0 | 0 | 0 | 0 | 0 | 0 | 0.681152 | 0.20787 | 0.34041 | 0.012258 | 0.37769 | 0.291204 | 0.013348 | 4.8663 | 2.32 | 3.7344 | 0.427 | 74.1978 |
| TJ | 2009 | 0.146415 | 0.092282 | 0.200549 | 0 | 0 | 0 | 0 | 0 | 0 | 0.762752 | 0.24308 | 0.44466 | 0.012885 | 0.406396 | 0.343471 | 0.034374 | 4.8453 | 2.37 | 5.645 | 0.49 | 90.1985 |
| TJ | 2010 | 0.197618 | 0.087517 | 0.307719 | 0 | 0 | 0 | 0 | 0 | 0 | 0.918083 | 0.290255 | 0.651142 | 0.014948 | 0.483757 | 0.412178 | 0.035695 | 3.3141 | 2.49 | 7.02279 | 0.51 | 108.4872 |
| TJ | 2011 | 0.127539 | 0.105927 | 0.14915 | 0 | 0 | 0 | 0 | 0 | 0 | 1.119099 | 0.339506 | 0.751067 | 0.015909 | 0.587802 | 0.515388 | 0.055454 | 3.1342 | 2.63 | 9.8019 | 0.454 | 130.5602 |
| TJ | 2012 | 0.283945 | 0.097254 | 0.470637 | 0 | 0 | 0 | 0 | 0 | 0 | 1.288518 | 0.392143 | 0.887131 | 0.017154 | 0.666368 | 0.612292 | 0.041094 | 7.542 | 2.8 | 7.2452 | 0.512 | 150.1633 |
| TJ | 2013 | 0.433889 | 0.131875 | 0.735903 | 0 | 0 | 0 | 0 | 0 | 0 | 1.437016 | 0.447043 | 1.01212 | 0.018845 | 0.727668 | 0.690503 | 0.035918 | 8.2315 | 2.96 | 5.49669 | 0.507 | 168.2897 |
| TJ | 2014 | 0.216907 | 0.13398 | 0.299835 | 0 | 0 | 0 | 0 | 0 | 0 | 1.572247 | 0.47387 | 1.165409 | 0.020153 | 0.776591 | 0.775503 | 0.032915 | 8.4736 | 2.96 | 4.9822 | 0.332 | 188.6676 |
| TJ | 2015 | 0.367947 | 0.150995 | 0.584899 | 0 | 1 | 0 | 0 | 0 | 0 | 1.653819 | 0.5257 | 1.306586 | 0.021051 | 0.77236 | 0.860408 | 0.0306 | 8.9389 | 3.08 | 2.2229 | 0.245 | 211.3444 |
| TJ | 2016 | 0.316764 | 0.103049 | 0.53048 | 0 | 1 | 0 | 0 | 0 | 0 | 1.7885 | 0.5636 | 1.27564 | 0.022022 | 0.800387 | 0.96613 | 0.030135 | 9.1615 | 3 | 1.14421 | 0.202 | 101.0045 |
| TJ | 2017 | 0.612641 | 0.225282 | 1 | 0 | 1 | 0 | 0 | 0 | 0 | 1.85492 | 0.57297 | 1.12889 | 0.0169 | 0.75936 | 1.07866 | 0.026714 | 9.6663 | 3.299118 | 6.229485 | 0.417956 | 186.4392 |
| SH | 2004 | 0.440906 | 0.119807 | 0.762004 | 0 | 0 | 0 | 0 | 0 | 0 | 0.816538 | 0.265691 | 0.308466 | 0.008345 | 0.389212 | 0.409726 | 0.000909 | 2.6508 | 2.12 | 8.4995 | 2.824 | 65.41 |
| SH | 2005 | 0.149896 | 0.099663 | 0.200128 | 0 | 0 | 0 | 0 | 0 | 0 | 0.924766 | 0.29795 | 0.01268 | 0.009026 | 0.43812 | 0.47762 | 0.001021 | 1.3728 | 2.27 | 4.7823 | 2.878 | 68.5 |
| SH | 2006 | 0.190584 | 0.079983 | 0.301184 | 0 | 0 | 0 | 0 | 0 | 0 | 1.071804 | 0.33752 | 0.39 | 0.009381 | 0.496995 | 0.550848 | 0.00124 | 1.464 | 2.45 | 16.6418 | 2.879 | 71.07 |
| SH | 2007 | 0.13176 | 0.102243 | 0.161276 | 0 | 0 | 0 | 0 | 0 | 0 | 1.266812 | 0.38733 | 0.44204 | 0.010184 | 0.557106 | 0.682111 | 0.007439 | 1.7783 | 2.46 | 7.6682 | 2.631 | 79.2 |
| SH | 2008 | 0.256888 | 0.094977 | 0.418799 | 0 | 0 | 0 | 0 | 0 | 0 | 1.42758 | 0.45772 | 0.482946 | 0.01118 | 0.623592 | 0.735043 | 0.006525 | 5.3061 | 2.53 | 8.1648 | 2.843 | 100.84 |
| SH | 2009 | 0.373814 | 0.146482 | 0.601145 | 0 | 0 | 0 | 0 | 0 | 0 | 1.528558 | 0.51732 | 0.46189 | 0.011382 | 0.593996 | 0.884715 | 0.027374 | 4.9847 | 2.81 | 28.4888 | 2.571 | 105.38 |
| SH | 2010 | 0.203963 | 0.152444 | 0.255482 | 0 | 0 | 0 | 0 | 0 | 0 | 1.687242 | 0.603686 | 0.531767 | 0.011415 | 0.713996 | 0.961831 | 0.024351 | 3.8584 | 2.81 | 28.67566 | 2.548 | 111.21 |
| SH | 2011 | 0.364447 | 0.146362 | 0.582532 | 0 | 0 | 0 | 0 | 0 | 0 | 1.919569 | 0.677711 | 0.506709 | 0.012494 | 0.795969 | 1.111106 | 0.035005 | 3.5045 | 3.11 | 28.193 | 2.742 | 126.01 |
| SH | 2012 | 0.291635 | 0.103176 | 0.480094 | 0 | 0 | 0 | 0 | 0 | 0 | 2.010133 | 0.738732 | 0.525438 | 0.01278 | 0.791277 | 1.206076 | 0.027584 | 7.1062 | 3.37 | 26.9187 | 3.081 | 151.85 |
| SH | 2013 | 0.605052 | 0.210103 | 1 | 0 | 0 | 0 | 0 | 0 | 0 | 2.160212 | 0.8557 | 0.564779 | 0.012928 | 0.802777 | 1.344507 | 0.031899 | 7.7999 | 3.56 | 22.11554 | 2.897 | 167.8 |
| SH | 2014 | 0.495226 | 0.137622 | 0.85283 | 0 | 0 | 0 | 0 | 0 | 0 | 2.356094 | 0.93035 | 0.601643 | 0.012426 | 0.816479 | 1.527189 | 0.031929 | 8.8929 | 3.66 | 26.7497 | 2.122 | 181.66 |
| SH | 2015 | 0.16405 | 0.097939 | 0.230162 | 0 | 1 | 0 | 0 | 0 | 0 | 2.512345 | 1.0132 | 0.63527 | 0.010978 | 0.794069 | 1.691452 | 0.044372 | 9.8996 | 3.73 | 25.9608 | 1.792 | 184.59 |
| SH | 2016 | 0.089921 | 0.068247 | 0.111595 | 0 | 1 | 0 | 0 | 0 | 0 | 2.8179 | 1.0947 | 0.67517 | 0.010947 | 0.799434 | 1.936234 | 0.058373 | 10.8905 | 3.82 | 36.84563 | 1.668 | 185.14 |
| SH | 2017 | 0.089354 | 0.110849 | 0.067858 | 0 | 1 | 0 | 0 | 0 | 0 | 3.0633 | 1.18303 | 0.72466 | 0.01108 | 0.93307 | 2.11915 | 0.056818 | 11.6763 | 3.983015 | 35.36581 | 2.690838 | 196.9199 |
| JS | 2004 | 0.125144 | 0.100834 | 0.149455 | 0 | 0 | 0 | 0 | 0 | 0 | 1.513678 | 0.489218 | 0.682759 | 0.136758 | 0.85369 | 0.52323 | 0.009371 | 1.9445 | 1.43 | 8.044 | 0.478 | 121.3783 |
| JS | 2005 | 0.316335 | 0.187961 | 0.444708 | 0 | 0 | 0 | 0 | 0 | 0 | 1.876933 | 0.57355 | 0.040016 | 0.146151 | 1.06584 | 0.664941 | 0.008392 | 1.1874 | 1.47 | 6.0633 | 0.512 | 131.8339 |
| JS | 2006 | 0.185603 | 0.159352 | 0.211854 | 0 | 0 | 0 | 0 | 0 | 0 | 2.196561 | 0.670619 | 1.007142 | 0.154505 | 1.245991 | 0.796065 | 0.007967 | 1.2993 | 1.59 | 9.1583 | 0.843 | 174.314 |
| JS | 2007 | 0.205714 | 0.164244 | 0.247184 | 0 | 0 | 0 | 0 | 0 | 0 | 2.629783 | 0.79859 | 1.226807 | 0.181631 | 1.469742 | 0.97841 | 0.030994 | 1.7809 | 1.65 | 21.0738 | 0.613 | 218.9206 |
| JS | 2008 | 0.188859 | 0.124545 | 0.253173 | 0 | 0 | 0 | 0 | 0 | 0 | 3.136064 | 0.99051 | 1.506045 | 0.210011 | 1.731209 | 1.194844 | 0.036553 | 3.1178 | 1.88 | 18.454 | 0.649 | 251.2001 |
| JS | 2009 | 0.435135 | 0.263441 | 0.60683 | 0 | 0 | 0 | 0 | 0 | 0 | 3.4912 | 1.148772 | 1.894988 | 0.226186 | 1.893912 | 1.371102 | 0.059481 | 3.4015 | 2.04 | 13.1945 | 0.739 | 253.2298 |
| JS | 2010 | 0.269625 | 0.148663 | 0.390588 | 0 | 0 | 0 | 0 | 0 | 0 | 4.090334 | 1.348232 | 2.318675 | 0.253959 | 2.175393 | 1.660982 | 0.056165 | 4.8359 | 2.07 | 13.10122 | 0.907 | 284.9777 |
| JS | 2011 | 0.13285 | 0.113879 | 0.151821 | 0 | 0 | 0 | 0 | 0 | 0 | 4.860426 | 1.584208 | 2.62994 | 0.306477 | 2.502378 | 2.051571 | 0.062955 | 3.2904 | 2.17 | 25.432 | 1.028 | 321.3173 |
| JS | 2012 | 0.098801 | 0.073219 | 0.124383 | 0 | 0 | 0 | 0 | 0 | 0 | 5.405822 | 1.821533 | 3.17072 | 0.341834 | 2.712192 | 2.351796 | 0.062399 | 4.9167 | 2.38 | 25.9215 | 1.127 | 357.5956 |
| JS | 2013 | 0.130026 | 0.159936 | 0.100116 | 0 | 0 | 0 | 0 | 0 | 0 | 5.916175 | 2.08782 | 3.598252 | 0.364608 | 2.909403 | 2.642164 | 0.058022 | 5.6782 | 2.49 | 20.43953 | 1.19 | 332.5922 |
| JS | 2014 | 0.105747 | 0.078828 | 0.132666 | 0 | 0 | 0 | 0 | 0 | 0 | 6.508832 | 2.34581 | 4.155275 | 0.363433 | 3.105747 | 3.039652 | 0.058694 | 6.1473 | 2.54 | 24.8722 | 0.868 | 281.7416 |
| JS | 2015 | 0.312277 | 0.193626 | 0.430929 | 0 | 1 | 0 | 0 | 0 | 0 | 7.011638 | 2.5877 | 4.590517 | 0.398794 | 3.204363 | 3.408481 | 0.056583 | 6.6981 | 2.57 | 12.469 | 0.754 | 242.7469 |
| JS | 2016 | 0.184436 | 0.15554 | 0.213333 | 0 | 1 | 0 | 0 | 0 | 0 | 7.7388 | 2.8707 | 4.93709 | 0.407848 | 3.385573 | 3.815196 | 0.051232 | 7.1773 | 2.66 | 9.48763 | 0.755 | 245.4296 |
| JS | 2017 | 0.162298 | 0.132585 | 0.192011 | 0 | 1 | 0 | 0 | 0 | 0 | 8.586976 | 3.17374 | 5.3277 | 0.40767 | 3.86549 | 4.316944 | 0.045394 | 7.7476 | 2.885809 | 23.59539 | 1.147368 | 352.9185 |
| ZJ | 2004 | 0.194537 | 0.13549 | 0.253584 | 0 | 0 | 1 | 0 | 0 | 0 | 1.16487 | 0.085545 | 0.120222 | 0.08141 | 0.625038 | 0.459472 | 0.007539 | 2.5507 | 0.99 | 4.5989 | 0.33 | 66.8128 |
| ZJ | 2005 | 0.369671 | 0.218872 | 0.52047 | 0 | 0 | 1 | 0 | 0 | 0 | 1.341768 | 0.46459 | 0.010278 | 0.014821 | 0.149436 | 0.130127 | 0.007435 | 2.0372 | 1.22 | 4.3295 | 0.504 | 77.2271 |
| ZJ | 2006 | 0.240158 | 0.123379 | 0.356938 | 0 | 0 | 1 | 0 | 0 | 0 | 1.571847 | 0.5358 | 0.146074 | 0.09251 | 0.851151 | 0.628186 | 0.007503 | 2.1817 | 1.43 | 7.6518 | 0.547 | 88.8935 |
| ZJ | 2007 | 0.144183 | 0.132376 | 0.15599 | 0 | 0 | 1 | 0 | 0 | 0 | 1.875373 | 0.62713 | 0.168413 | 0.098602 | 1.015425 | 0.761346 | 0.024945 | 3.0178 | 1.5 | 9.7116 | 0.543 | 103.6576 |
| ZJ | 2008 | 0.109261 | 0.087371 | 0.13115 | 0 | 0 | 1 | 0 | 0 | 0 | 2.146269 | 0.75333 | 0.19805 | 0.109596 | 1.156742 | 0.879931 | 0.026908 | 4.0991 | 1.61 | 10.9616 | 0.675 | 100.7294 |
| ZJ | 2009 | 0.188759 | 0.195597 | 0.181921 | 0 | 0 | 1 | 0 | 0 | 0 | 2.299824 | 0.86223 | 0.229165 | 0.116308 | 1.186016 | 0.997501 | 0.092834 | 4.3041 | 1.73 | 7.1235 | 0.683 | 99.3974 |
| ZJ | 2010 | 0.165536 | 0.10835 | 0.222721 | 0 | 0 | 1 | 0 | 0 | 0 | 2.72268 | 1.01632 | 1.24881 | 0.13607 | 1.41213 | 1.17448 | 0.072737 | 2.9408 | 1.78 | 10.59284 | 0.866 | 110.0175 |
| ZJ | 2011 | 0.306578 | 0.236493 | 0.376662 | 0 | 0 | 1 | 0 | 0 | 0 | 3.20001 | 1.19306 | 1.42901 | 0.15806 | 1.64042 | 1.40154 | 0.071301 | 5.2837 | 1.85 | 8.5523 | 0.529 | 116.6601 |
| ZJ | 2012 | 0.281012 | 0.180474 | 0.381551 | 0 | 0 | 1 | 0 | 0 | 0 | 3.46063 | 1.35463 | 1.7096 | 0.16695 | 1.73124 | 1.56244 | 0.069063 | 5.7737 | 2.08 | 7.974 | 0.61 | 130.6926 |
| ZJ | 2013 | 0.140989 | 0.1454 | 0.136579 | 0 | 0 | 1 | 0 | 0 | 0 | 3.75685 | 1.59708 | 2.01941 | 0.17846 | 1.84467 | 1.73372 | 0.078741 | 6.4156 | 2.16 | 5.26234 | 0.992 | 141.5898 |
| ZJ | 2014 | 0.169502 | 0.165774 | 0.173229 | 0 | 0 | 1 | 0 | 0 | 0 | 4.01535 | 1.78353 | 2.35548 | 0.17793 | 1.91527 | 1.92215 | 0.075269 | 7.0156 | 2.26 | 4.8696 | 0.725 | 157.9725 |
| ZJ | 2015 | 0.438311 | 0.237234 | 0.639389 | 1 | 1 | 1 | 1 | 0 | 0 | 4.288649 | 1.9785 | 2.666472 | 0.18328 | 1.97071 | 2.13466 | 0.08328 | 7.5002 | 2.36 | 3.5682 | 0.644 | 169.6024 |
| ZJ | 2016 | 0.340871 | 0.133536 | 0.548206 | 1 | 1 | 1 | 0 | 1 | 0 | 4.7251 | 2.1971 | 2.9571 | 0.196518 | 2.119461 | 2.409157 | 0.066475 | 8.3408 | 2.43 | 3.5327 | 0.61 | 175.7748 |
| ZJ | 2017 | 0.143922 | 0.146333 | 0.141512 | 1 | 1 | 1 | 0 | 0 | 1 | 5.176826 | 2.43085 | 3.1696 | 0.201743 | 2.247152 | 2.727931 | 0.042565 | 8.7811 | 2.650809 | 7.752952 | 0.905434 | 184.2077 |
| AH | 2004 | 0.214023 | 0.098631 | 0.329415 | 0 | 0 | 1 | 0 | 0 | 0 | 0.47593 | 0.15574 | 0.19353 | 0.095051 | 0.18449 | 0.196392 | 0.00958 | 1.1385 | 0.8 | 2.6748 | 0.101 | 5.4669 |
| AH | 2005 | 0.21938 | 0.231051 | 0.207708 | 0 | 0 | 1 | 0 | 0 | 0 | 0.53502 | 0.17767 | 0.003292 | 0.09665 | 0.22459 | 0.21378 | 0.00952 | 0.6273 | 0.85 | 5.0616 | 0.184 | 6.8845 |
| AH | 2006 | 0.1904 | 0.112313 | 0.268488 | 0 | 0 | 1 | 0 | 0 | 0 | 0.61125 | 0.20565 | 0.35336 | 0.101103 | 0.271118 | 0.239029 | 0.008506 | 0.7213 | 0.97 | 1.5066 | 0.168 | 13.9354 |
| AH | 2007 | 0.474761 | 0.283478 | 0.666045 | 0 | 0 | 1 | 0 | 0 | 0 | 0.73609 | 0.24519 | 0.50875 | 0.120018 | 0.337096 | 0.278978 | 0.039797 | 0.854 | 0.98 | 2.0053 | 0.226 | 29.9892 |
| AH | 2008 | 0.214179 | 0.187936 | 0.240422 | 0 | 0 | 1 | 0 | 0 | 0 | 0.88517 | 0.30452 | 0.679995 | 0.14181 | 0.41374 | 0.33187 | 0.031932 | 2.2808 | 1.11 | 3.8966 | 0.183 | 34.8988 |
| AH | 2009 | 0.264414 | 0.149595 | 0.379233 | 0 | 0 | 1 | 0 | 0 | 0 | 1.006282 | 0.35278 | 0.79455 | 0.149545 | 0.490522 | 0.366215 | 0.066656 | 2.5098 | 1.35 | 8.9559 | 0.156 | 38.8416 |
| AH | 2010 | 0.331928 | 0.171486 | 0.492371 | 0 | 0 | 1 | 0 | 0 | 0 | 1.22634 | 0.41515 | 1.18494 | 0.1279 | 0.63911 | 0.41433 | 0.048642 | 3.9741 | 1.32 | 3.3363 | 0.207 | 50.1446 |
| AH | 2011 | 0.633013 | 0.272697 | 0.993329 | 0 | 0 | 1 | 0 | 0 | 0 | 1.51103 | 0.49006 | 1.21263 | 0.20203 | 0.82264 | 0.48636 | 0.066428 | 1.6018 | 1.4 | 6.9982 | 0.248 | 66.2887 |
| AH | 2012 | 0.459199 | 0.140501 | 0.777897 | 0 | 0 | 1 | 0 | 0 | 0 | 1.72121 | 0.56856 | 1.5055 | 0.21787 | 0.9404 | 0.56294 | 0.060246 | 4.2787 | 1.64 | 4.8961 | 0.272 | 86.3811 |
| AH | 2013 | 0.206569 | 0.163272 | 0.249866 | 0 | 0 | 1 | 0 | 0 | 0 | 1.90389 | 0.70447 | 1.82511 | 0.23481 | 1.0404 | 0.62868 | 0.063427 | 4.7235 | 1.83 | 6.23781 | 0.233 | 106.8772 |
| AH | 2014 | 0.109386 | 0.091363 | 0.127408 | 0 | 0 | 1 | 0 | 0 | 0 | 2.08488 | 0.7957 | 2.12563 | 0.23924 | 1.1204 | 0.72524 | 0.072558 | 5.0271 | 1.89 | 3.9967 | 0.229 | 123.3978 |
| AH | 2015 | 0.147911 | 0.202478 | 0.093344 | 1 | 1 | 1 | 1 | 0 | 0 | 2.200563 | 0.8908 | 2.39656 | 0.24567 | 1.13423 | 0.82066 | 0.073411 | 5.6659 | 1.96 | 2.6001 | 0.203 | 136.1945 |
| AH | 2016 | 0.125346 | 0.120935 | 0.129757 | 1 | 1 | 1 | 0 | 1 | 0 | 2.4408 | 1 | 2.65774 | 0.25677 | 1.16666 | 0.98836 | 0.061731 | 6.1038 | 1.97 | 2.21941 | 0.237 | 147.6712 |
| AH | 2017 | 0.406917 | 0.321078 | 0.492756 | 1 | 1 | 1 | 0 | 0 | 1 | 2.7018 | 1.11926 | 2.92751 | 0.26117 | 1.348661 | 1.14204 | 0.037134 | 6.5444 | 2.064853 | 5.620223 | 0.280382 | 136.7214 |
| HN | 2004 | 0.212811 | 0.188692 | 0.23693 | 0 | 0 | 1 | 0 | 0 | 0 | 0.566437 | 0.21629 | 0.198129 | 0.10411 | 0.220445 | 0.241882 | 0.01303 | 1.421 | 0.66 | 1.3758 | 0.027 | 14.1806 |
| HN | 2005 | 0.161556 | 0.166604 | 0.156508 | 0 | 0 | 1 | 0 | 0 | 0 | 0.65961 | 0.24743 | 0.003879 | 0.110065 | 0.261257 | 0.288288 | 0.012511 | 0.9288 | 0.68 | 0.3214 | 0.037 | 20.7235 |
| HN | 2006 | 0.217791 | 0.191073 | 0.244508 | 0 | 0 | 1 | 0 | 0 | 0 | 0.772232 | 0.28694 | 0.324239 | 0.12722 | 0.320874 | 0.324138 | 0.010765 | 0.8568 | 0.7 | 0.2533 | 0.021 | 25.9335 |
| HN | 2007 | 0.399136 | 0.280991 | 0.517281 | 0 | 0 | 1 | 0 | 0 | 0 | 0.948599 | 0.34192 | 0.429436 | 0.162648 | 0.401068 | 0.384883 | 0.038143 | 1.1113 | 0.78 | 0.4156 | 0.034 | 32.7051 |
| HN | 2008 | 0.278107 | 0.156441 | 0.399773 | 0 | 0 | 1 | 0 | 0 | 0 | 1.162761 | 0.42226 | 0.564969 | 0.18924 | 0.508461 | 0.46506 | 0.034774 | 2.5428 | 0.98 | 3.1642 | 0.04 | 40.0515 |
| HN | 2009 | 0.143714 | 0.159683 | 0.127745 | 0 | 0 | 1 | 0 | 0 | 0 | 1.315627 | 0.49137 | 0.688 | 0.196969 | 0.575909 | 0.542749 | 0.055635 | 2.8722 | 1.18 | 3.3634 | 0.036 | 45.9787 |
| HN | 2010 | 0.114811 | 0.104915 | 0.124708 | 0 | 0 | 1 | 0 | 0 | 0 | 1.59021 | 0.57753 | 0.98211 | 0.23394 | 0.73136 | 0.64291 | 0.062076 | 4.9623 | 1.16 | 6.61671 | 0.061 | 51.8441 |
| HN | 2011 | 0.140804 | 0.197152 | 0.084456 | 0 | 0 | 1 | 0 | 0 | 0 | 1.96352 | 0.6809 | 1.14315 | 0.27337 | 0.93247 | 0.75768 | 0.095357 | 1.9888 | 1.19 | 4.9332 | 0.055 | 61.5031 |
| HN | 2012 | 0.113968 | 0.104359 | 0.123578 | 0 | 0 | 1 | 0 | 0 | 0 | 2.21542 | 0.78549 | 1.45766 | 0.30042 | 1.05064 | 0.86436 | 0.072024 | 4.5477 | 1.3 | 4.8174 | 0.069 | 72.8034 |
| HN | 2013 | 0.324565 | 0.20927 | 0.439861 | 0 | 0 | 1 | 0 | 0 | 0 | 2.45017 | 0.95095 | 1.83814 | 0.30992 | 1.15174 | 0.98851 | 0.077809 | 4.9583 | 1.33 | 5.94177 | 0.105 | 87.0482 |
| HN | 2014 | 0.209519 | 0.189877 | 0.22916 | 0 | 0 | 1 | 0 | 0 | 0 | 2.70485 | 1.07235 | 2.19508 | 0.31488 | 1.24819 | 1.14178 | 0.064318 | 5.3816 | 1.36 | 6.2307 | 0.069 | 102.6585 |
| HN | 2015 | 0.14895 | 0.138655 | 0.159246 | 1 | 1 | 1 | 1 | 0 | 0 | 2.890221 | 1.2024 | 2.59543 | 0.33316 | 1.29554 | 1.27602 | 0.052333 | 6.0105 | 1.43 | 4.027 | 0.047 | 115.6441 |
| HN | 2016 | 0.222049 | 0.200184 | 0.243914 | 1 | 1 | 1 | 0 | 1 | 0 | 3.1551 | 1.3437 | 2.76884 | 0.35784 | 1.3181 | 1.44853 | 0.050366 | 6.5487 | 1.5 | 1.82621 | 0.057 | 128.5208 |
| HN | 2017 | 0.277976 | 0.17046 | 0.385491 | 1 | 1 | 1 | 0 | 0 | 1 | 3.390296 | 1.48549 | 3.19592 | 0.369 | 1.41455 | 1.675511 | 0.048342 | 7.1387 | 1.573676 | 5.803014 | 0.076522 | 116.9662 |
| SD | 2004 | 0.252686 | 0.122866 | 0.382506 | 0 | 0 | 1 | 0 | 0 | 0 | 1.502184 | 0.52905 | 0.762904 | 0.177845 | 0.847869 | 0.47647 | 0.007081 | 1.8683 | 0.95 | 3.8313 | 0.461 | 87.0064 |
| SD | 2005 | 0.119697 | 0.116124 | 0.123271 | 0 | 0 | 1 | 0 | 0 | 0 | 1.836687 | 0.61669 | 0.019856 | 0.196351 | 1.047862 | 0.592474 | 0.006645 | 1.0523 | 1.05 | 5.9385 | 0.657 | 89.7072 |
| SD | 2006 | 0.11567 | 0.106448 | 0.124893 | 0 | 0 | 1 | 0 | 0 | 0 | 2.190019 | 0.72171 | 1.11361 | 0.21389 | 1.257403 | 0.718726 | 0.006147 | 1.2231 | 1.07 | 6.2841 | 0.69 | 100.0069 |
| SD | 2007 | 0.14874 | 0.209882 | 0.087598 | 0 | 0 | 1 | 0 | 0 | 0 | 2.577691 | 0.86075 | 1.253702 | 0.250914 | 1.464753 | 0.862024 | 0.000704 | 1.4418 | 1.21 | 14.5022 | 0.639 | 110.1159 |
| SD | 2008 | 0.118746 | 0.116465 | 0.121026 | 0 | 0 | 1 | 0 | 0 | 0 | 3.093328 | 1.06588 | 1.543593 | 0.300265 | 1.757198 | 1.035864 | 0.01111 | 3.1798 | 1.4 | 18.5503 | 0.596 | 82.0246 |
| SD | 2009 | 0.325866 | 0.21748 | 0.434252 | 0 | 0 | 1 | 0 | 0 | 0 | 3.389665 | 1.2363 | 1.54391 | 0.322664 | 1.890183 | 1.176818 | 0.05334 | 3.4477 | 1.53 | 12.0687 | 0.624 | 80.1007 |
| SD | 2010 | 0.209494 | 0.194979 | 0.224008 | 0 | 0 | 1 | 0 | 0 | 0 | 3.94162 | 1.42116 | 2.32791 | 0.35883 | 2.13989 | 1.4429 | 0.055616 | 3.1748 | 1.72 | 16.09652 | 0.738 | 91.6833 |
| SD | 2011 | 0.15579 | 0.150873 | 0.160708 | 0 | 0 | 1 | 0 | 0 | 0 | 4.54292 | 1.66759 | 2.59285 | 0.39738 | 2.40374 | 1.7418 | 0.058968 | 2.4141 | 1.86 | 18.3306 | 0.098 | 111.6022 |
| SD | 2012 | 0.224921 | 0.208797 | 0.241045 | 0 | 0 | 1 | 0 | 0 | 0 | 5.00132 | 1.91753 | 3.03198 | 0.42817 | 2.57357 | 1.99958 | 0.054718 | 5.0097 | 2.04 | 15.9973 | 0.142 | 123.5267 |
| SD | 2013 | 0.296293 | 0.195293 | 0.397294 | 0 | 0 | 1 | 0 | 0 | 0 | 5.46843 | 2.22948 | 3.58759 | 0.47426 | 2.74225 | 2.25192 | 0.055454 | 5.5174 | 2.13 | 21.50353 | 1.048 | 140.5315 |
| SD | 2014 | 0.262468 | 0.13404 | 0.390897 | 0 | 0 | 1 | 0 | 0 | 0 | 5.94266 | 2.51115 | 4.15991 | 0.47984 | 2.87881 | 2.58401 | 0.055622 | 6.0303 | 2.19 | 16.002 | 0.989 | 151.9511 |
| SD | 2015 | 0.131078 | 0.132436 | 0.129719 | 1 | 1 | 1 | 1 | 0 | 0 | 6.300233 | 2.7761 | 4.738145 | 0.497908 | 2.94859 | 2.853735 | 0.055841 | 6.6189 | 2.27 | 8.5804 | 0.849 | 163.009 |
| SD | 2016 | 0.11631 | 0.108583 | 0.124037 | 1 | 1 | 1 | 0 | 1 | 0 | 6.8024 | 3.0646 | 5.23645 | 0.492913 | 3.041003 | 3.166903 | 0.042612 | 7.0509 | 2.34 | 9.47435 | 0.418 | 168.2556 |
| SD | 2017 | 0.143448 | 0.202893 | 0.084002 | 1 | 1 | 1 | 0 | 0 | 1 | 7.263415 | 3.3649 | 5.52027 | 0.487674 | 3.292512 | 3.487632 | 0.039673 | 7.6723 | 2.513824 | 17.73739 | 0.740243 | 167.6075 |
| GD | 2004 | 0.112515 | 0.107083 | 0.117947 | 0 | 0 | 1 | 0 | 0 | 0 | 1.886462 | 0.685203 | 0.602553 | 0.121984 | 0.928073 | 0.836405 | 0.004618 | 2.9039 | 1.12 | 8.7203 | 0.418 | 100.1158 |
| GD | 2005 | 0.322852 | 0.229587 | 0.416117 | 0 | 0 | 1 | 0 | 0 | 0 | 2.255737 | 0.79155 | 0.031829 | 0.142827 | 1.13566 | 0.97725 | 0.00475 | 1.5036 | 1.09 | 11.3983 | 0.537 | 123.6391 |
| GD | 2006 | 0.218683 | 0.213283 | 0.224084 | 0 | 0 | 1 | 0 | 0 | 0 | 2.658776 | 0.91943 | 0.813237 | 0.153217 | 1.346977 | 1.158582 | 0.00541 | 1.6338 | 1.18 | 5.1799 | 0.668 | 145.1065 |
| GD | 2007 | 0.146216 | 0.148521 | 0.143912 | 0 | 0 | 1 | 0 | 0 | 0 | 3.177701 | 1.07313 | 0.959695 | 0.169557 | 1.600461 | 1.407683 | 0.018078 | 1.7131 | 1.27 | 7.0535 | 0.557 | 171.2603 |
| GD | 2008 | 0.221387 | 0.221248 | 0.221526 | 0 | 0 | 1 | 0 | 0 | 0 | 3.679671 | 1.29866 | 1.116506 | 0.197305 | 1.85022 | 1.632146 | 0.028011 | 4.3097 | 1.37 | 9.2655 | 0.805 | 191.6703 |
| GD | 2009 | 0.302819 | 0.21607 | 0.389567 | 0 | 0 | 1 | 0 | 0 | 0 | 3.949252 | 1.48918 | 1.02301 | 0.201027 | 1.933828 | 1.814397 | 0.057478 | 4.5631 | 1.65 | 7.3403 | 0.918 | 195.346 |
| GD | 2010 | 0.263162 | 0.14775 | 0.378573 | 0 | 0 | 1 | 0 | 0 | 0 | 4.601306 | 1.741466 | 1.25993 | 0.228695 | 2.301453 | 2.146272 | 0.058686 | 3.3697 | 1.76 | 7.76901 | 1.059 | 202.6098 |
| GD | 2011 | 0.124366 | 0.118694 | 0.130037 | 0 | 0 | 1 | 0 | 0 | 0 | 5.321028 | 2.024672 | 1.6844 | 0.26652 | 2.644738 | 2.40977 | 0.079465 | 2.6959 | 1.96 | 9.7786 | 0.211 | 217.9836 |
| GD | 2012 | 0.11584 | 0.111179 | 0.120501 | 0 | 0 | 1 | 0 | 0 | 0 | 5.706792 | 2.267711 | 1.930753 | 0.284891 | 2.78253 | 2.639371 | 0.069289 | 5.8895 | 2.17 | 7.6374 | 0.198 | 235.4911 |
| GD | 2013 | 0.143806 | 0.206076 | 0.081536 | 0 | 0 | 1 | 0 | 0 | 0 | 6.216397 | 2.545393 | 2.285853 | 0.304751 | 2.942749 | 2.968897 | 0.08324 | 6.598 | 2.31 | 7.62387 | 1.047 | 249.521 |
| GD | 2014 | 0.115286 | 0.113561 | 0.117012 | 0 | 0 | 1 | 0 | 0 | 0 | 6.779224 | 2.84711 | 2.592809 | 0.316667 | 3.134577 | 3.32798 | 0.096653 | 7.2211 | 2.37 | 6.4423 | 0.833 | 268.7144 |
| GD | 2015 | 0.323009 | 0.238904 | 0.407115 | 1 | 1 | 1 | 1 | 0 | 0 | 7.281255 | 3.1518 | 2.592809 | 0.334482 | 3.251149 | 3.695624 | 0.217053 | 7.968 | 2.47 | 5.2251 | 0.72 | 268.7546 |
| GD | 2016 | 0.227179 | 0.235263 | 0.219094 | 1 | 1 | 1 | 0 | 1 | 0 | 8.0855 | 3.4739 | 3.29473 | 0.369358 | 3.437246 | 4.144601 | 0.075443 | 8.4444 | 2.56 | 4.75105 | 0.769 | 233.49 |
| GD | 2017 | 0.145147 | 0.150791 | 0.139503 | 1 | 1 | 1 | 0 | 0 | 1 | 8.970523 | 3.82001 | 3.77617 | 0.37924 | 3.85986 | 4.74883 | 0.056419 | 9.1022 | 2.544853 | 8.472747 | 0.903456 | 275.7493 |
| CQ | 2004 | 0.224709 | 0.236074 | 0.213343 | 0 | 0 | 0 | 0 | 0 | 0 | 0.304803 | 0.106833 | 0.162192 | 0.042805 | 0.138684 | 0.123314 | 0.011151 | 1.4665 | 0.88 | 5.0309 | 0.154 | 4.0508 |
| CQ | 2005 | 0.314257 | 0.230423 | 0.398091 | 0 | 0 | 0 | 0 | 0 | 0 | 0.348622 | 0.122781 | 0.00283 | 0.04634 | 0.157766 | 0.144516 | 0.010702 | 1.1245 | 1.04 | 0.3409 | 0.211 | 5.1575 |
| CQ | 2006 | 0.278381 | 0.165298 | 0.391465 | 0 | 0 | 0 | 0 | 0 | 0 | 0.392967 | 0.143151 | 0.245184 | 0.038638 | 0.188821 | 0.165508 | 0.010376 | 1.3359 | 0.94 | 0.348 | 0.193 | 6.9595 |
| CQ | 2007 | 0.135394 | 0.133685 | 0.137103 | 0 | 0 | 0 | 0 | 0 | 0 | 0.470401 | 0.171112 | 0.316151 | 0.048239 | 0.22024 | 0.201922 | 0.046955 | 1.3139 | 1 | 0.6716 | 0.257 | 10.2857 |
| CQ | 2008 | 0.11975 | 0.111943 | 0.127556 | 0 | 0 | 0 | 0 | 0 | 0 | 0.582986 | 0.214712 | 0.404525 | 0.05754 | 0.26133 | 0.264116 | 0.033167 | 2.5596 | 1.04 | 0.6895 | 0.24 | 24.5196 |
| CQ | 2009 | 0.153658 | 0.224168 | 0.083148 | 0 | 0 | 0 | 0 | 0 | 0 | 0.657696 | 0.251502 | 0.531792 | 0.06068 | 0.297333 | 0.299683 | 0.035736 | 2.825 | 1.22 | 1.8598 | 0.272 | 33.7577 |
| CQ | 2010 | 0.12072 | 0.122873 | 0.118568 | 0 | 0 | 0 | 0 | 0 | 0 | 0.789424 | 0.287804 | 0.69348 | 0.068539 | 0.435641 | 0.285244 | 0.029769 | 3.6496 | 1.27 | 2.09699 | 0.276 | 30.4264 |
| CQ | 2011 | 0.340292 | 0.268807 | 0.411777 | 0 | 0 | 0 | 0 | 0 | 0 | 1.001113 | 0.34878 | 0.76318 | 0.084452 | 0.55428 | 0.362381 | 0.072363 | 2.4411 | 1.28 | 1.8197 | 0.297 | 58.2575 |
| CQ | 2012 | 0.241122 | 0.254826 | 0.227417 | 0 | 0 | 0 | 0 | 0 | 0 | 1.1459 | 0.396119 | 0.938 | 0.094001 | 0.617233 | 0.434666 | 0.067908 | 4.8702 | 1.4 | 3.0271 | 0.326 | 35.2418 |
| CQ | 2013 | 0.144388 | 0.151016 | 0.137759 | 0 | 0 | 0 | 0 | 0 | 0 | 1.265669 | 0.50558 | 1.120503 | 0.100268 | 0.639792 | 0.525609 | 0.082997 | 5.5112 | 1.38 | 3.5997 | 0.324 | 41.4353 |
| CQ | 2014 | 0.241138 | 0.266448 | 0.215829 | 0 | 0 | 0 | 0 | 0 | 0 | 1.42654 | 0.57107 | 1.322375 | 0.106103 | 0.653186 | 0.667251 | 0.052992 | 5.6039 | 1.42 | 3.7336 | 0.311 | 42.3348 |
| CQ | 2015 | 0.362445 | 0.286205 | 0.438685 | 0 | 1 | 0 | 0 | 0 | 0 | 1.571727 | 0.6424 | 1.548033 | 0.115015 | 0.707182 | 0.749775 | 0.077511 | 6.0296 | 1.57 | 2.9746 | 0.28 | 37.7183 |
| CQ | 2016 | 0.292346 | 0.179491 | 0.405201 | 0 | 1 | 0 | 0 | 0 | 0 | 1.7741 | 0.7271 | 1.59318 | 0.130324 | 0.775516 | 0.850036 | 0.066968 | 6.3717 | 1.72 | 0.63069 | 0.21 | 26.263 |
| CQ | 2017 | 0.14171 | 0.145269 | 0.13815 | 0 | 1 | 0 | 0 | 0 | 0 | 1.942473 | 0.80677 | 1.7537 | 0.12761 | 0.85846 | 0.9564 | 0.066409 | 7.0036 | 1.701397 | 3.022699 | 0.365801 | 48.51703 |
